# Sex difference and risk factors in burden of urogenital congenital anomalies from 1990 to 2019

**DOI:** 10.1038/s41598-023-40939-3

**Published:** 2023-08-22

**Authors:** Xiaoyu Huang, Jianming Tang, Mao Chen, Ya Xiao, Fangyi Zhu, Liying Chen, Xiaoyu Tian, Li Hong

**Affiliations:** 1 Department of Gynecology and Obstetrics, Renmin Hospital of Wuhan University, Wuhan, China; 2 Pelvic Floor Research Centre of Hubei Province, Renmin Hospital of Wuhan University, Wuhan, China

**Keywords:** Urogenital diseases, Urogenital reproductive disorders, Urology, Paediatric urology, Urogenital diseases

## Abstract

Urogenital congenital anomalies (UCAs) is defined as “any live-birth with a urinary or genital condition” and affects millions of men and women worldwide. However, sex differences and related environmental risk factors in UCAs burden on a global scale have not been assessed. Using data from the Global Burden of Diseases, Injuries, and Risk Factors Study (GBD) 2019, we estimated prevalence, incidence, mortality and disability-adjusted life years (DALYs) of UCAs from 1990 to 2019 by sex, region, and socio-demographic Index (SDI) in 204 countries and territories. The disease burden of UCAs was also estimated attributable to each risk factor were estimated according to risk exposure. In 2019, UCAs caused 10,200 all-ages deaths (95% UI 7550–13,400). The combined global incidence rate was 8.38 per 1000 (95% UI 5.88–12.0) live births. The ASIR increased slightly, while the ASDR decreased from 1990 to 2019.The UCAs burden varies greatly depending on the development level and geographical location. The UCAs burden was significantly higher in men than in women, and the sex differences showed an enlarging trend. Health risks and issues, including pollution, child and maternal malnutrition, diet habits, unsafe sanitation and water source, were detected to be positively related to UCAs burden. Albeit the age-standardised prevalence, mortality, incidence, and DALYs of UCAs have decreased, they still cause a public health challenge worldwide. The high deaths and DALYs rates in low and low-middle SDI countries highlight the urgent need for improved preventive, diagnostic, and therapeutic measures. Global strategies for enhancing water safety, reducing pollution, and healthy diets are crucial steps in reducing the burden of UCAs.

## Introduction

Urogenital congenital anomalies (UCAs) are among the most common organ system abnormalities in the neonate. Here, UCAs are characterized as “any live-birth with a urinary or genital condition”. UCAs are often discovered in the prenatal or immediately postnatal period, with a substantial percentage being found in older children with varied degrees of severity^1^. UCAs often present as incontinence, recurrent urinary tract infections, atypical genitalia or other abdominal issues due to congenital anomalies of the urinary tract^2^. Unfortunately, the impairments due to UCAs are not only serious causes of death after birth but also lead to poor life prospects. Congenital abnormalities of the kidney and urinary tract are also one of the main causes of end-stage renal disease^3^. As a result, patients with UCAs might require urgent medical or surgical care, or lifelong hormone replacement or monitoring for gonadal cancer^4^. Meanwhile, it cannot be ignored that the malformations of the genital condition even lead to severe public health, financial, and psychological burdens. However, the latest spatial patterns and temporal trends of UCA burden at the global, regional, and national levels remain still lacking.

The Global Burden of Disease (GBD) study 2019 is the latest systematic epidemiological study of global diseases and their risk factors, using all known data on diseases or disorders from administrative and community survey sources to establish and analyze correlations to assess trends, thereby rendering the data more systematic and reliable^5,6^.

In the current report we present global, regional, and national-level estimates of prevalence, incidence, mortality and disability-adjusted life years (DALYs) of UCAs in the general population. Estimates are reported in terms of numbers (count) and age-standardised rates by sex, for 204 countries and territories, from 1990 to 2019. We also highlight the relative association of pollution, child and maternal malnutrition, diet habits, unsafe sanitation and water source and burden of UCAs. This study is the first research to describe the global landscape of UCAs and provides a theoretical basis for the UCAs prevention.

## Methods

### Data source

The analytical framework for the GBD 2019 and estimation methods of UCA burden have been delineated in previous studies^5,7^. Every step of this research adhered to the Guidelines for Accurate and Transparent Health Estimates Reporting^8^. The source data, including case definitions, epidemiological estimators, exposures, risk estimates, are freely available at the Global Health Data Exchange (GHDx). The spectrum of congenital malformations of the genitalia is broad, in this study, UCAs were defined as any live births with a urinary or genital condition, including congenital malformation of the collecting system, ureter, bladder, and kidney; bladder exstrophy and epispadias; hypospadias; ambiguous or indeterminate sex; and other genital malformations according to GBD (https://www.healthdata.org)^9^. The systematic collection of epidemiological UCA burden was conducted by GBD 2019 in three stages involving electronic searches of the peer-reviewed literature in the Embase and PubMed databases; the gray literature; and expert consultations to report estimates of prevalence, incidence, remission, and excess mortality for UCAs. To describe the disease burden of UCAs in different geographic units, the 204 countries and territories were separated into 21 GBD regions according to a geographic hierarchy. Socio-demographic Index (SDI) is a composite indicator of a country’s lag-distributed income per capita, average years of schooling and the total fertility rate in females under the age of 25 years. The SDI, ranging from 0 to 100, indicates socio-demographic development by incorporating lagged distributed income per capita, average years of education, and total fertility rate. We used the SDI to classify the 204 countries and territories were further classified into five regions in terms of their corresponding SDI in 2019, namely low, low-middle, middle, high-middle and high SDI (Socio-demographic Index) regions^5,10^.

We also collected summary exposure value (SEV) of risk factors and human development index (HDI) at the national level from the World Bank. GBD risk factors are described in Supplementary Table 1. The Human Development Index (HDI) is one of the accepted indicators for determining progress, living conditions, and human development in different countries. This index includes socioeconomic variables, including literacy, life expectancy, and income that affect health. HDI information was derived from the UNITED NATIONS DEVELOPMENT PROGRAMME website (http://hdr.undp.org/en/data). The HDI scale ranges from 0 to 1.0, with 1.0 representing the highest level of human development. Very High Human Development (0.8–1.0), High Human Development (0.7–0.79), Medium Human Development (0.55–0.70), and Low Human Development (below 0.55) are the 4 levels of HDI.

### Statistical analysis

Based on the GBD global reference population, we applied the age-standardized incidence rate (ASIR), and age-standardized deaths rate (ASDR) to quantify the UCA burden by location, sex, and SDI from 1990 to 2019. The 95% uncertainty intervals (UIs) for every metric in the GBD study were produced based on the 25th and 975th ordered values of 1000 random draws of the posterior distribution^11^. We further used the average annual percentage change (AAPC) to describe the temporal trend in various age-standardized rates (ASRs) of the UCA burden from 1990 to 2019. We performed a regression model fitting the natural logarithm of the ASR with the calendar year, namely, ln (ASR) = α + β* calendar year + ε, to estimate the AAPC with its 95% confidence interval (CI) based on the formula of 100 × (exp (β) − 1)^12,13^. Additionally, to explore the influential factors for EAPCs in UCAs, we evaluated the correlation between EAPCs and baseline burden in 1990 as well as SDI in 2019 using the Spearman rank correlation test at the national level. The ASRs of UCAs in 1990 could serve as the disease burden at baseline, and the SDI in 2019 is a composite indicator to reflect the availability and level of health care in different countries or regions^14,15^. All statistical analyses in this study were performed using R program version 4.0.3 (https://www.R-project.org/). A two-sided p-value of < 0.05 was considered statistically significant.

### Ethics approval and consent to participate

Ethics approval was exempted by the Ethics Committee of Wuhan University, because the GBD is a publicly available database and all participants’ data were anonymous.

## Results

### The global burden and temporal trend in UCAs with sex differences

In 2019, urogenital congenital anomalies caused 10,200 all-ages deaths (95% UI 7550–13 400), with a combined global incidence rate of 8.38 per 1000 (95% UI 5.88–12.0) live births and a total of 6.28 million individuals living with such anomalies (95% UI 4.98–7.72) in 2019. The age-standardized incidence rate (ASIR) per 100,000 population exhibited a slight increase from 16.91 (95% UI 11.88–24.11) in 1990 to 17.52 (95% UI 12.28–25.05) in 2019 (Supplementary Table 2). Conversely, the age-standardized death rate (ASDR) per 100,000 population experienced a slight decrease from 0.23 (95% UI 0.14–0.36) in 1990 to 0.15 (95% UI 0.11–0.20) in 2019. Additionally, there was 16.4 (95% UI 12.4–21.0) disability-adjusted life years (DALYs) rate per 10,000 population in 2019, showing a decrease of 13.6% since 2010 (Table 1, Supplementary Tables 2, 3).

**Table 1:** The number of cases, rate and age-standardized rate per 100,000 people of DALYs for both sexes, and female and male urogenital congenital anomalies in 2010 and 2019. DALYs, disability adjusted life years.

Characteristics	2010			2019		
	DALYs (millions)	Rate (per 100,000)	ASR of DALYs (per 10,000)	DALYs (millions)	Rate (per 100,000)	ASR of DALYs (per 10,000)
Global-both sex	1.475 (0.920–2.24)	27.58 (17.20–41.88)	22.94 (14.37–34.73)	1.094 (0.832–1.408)	14.14 (10.76–18.20)	16.41 (12.43–21.05)
Global-female	0.592 (0.363–1.127)	22.27 (13.66–43.43)	10.03 (11.72–36.10)	0.438 (0.302–0.604)	11.35 (7.83–15.66)	13.48 (9.24–18.72)
Global-male	0.884 (0.519–1.479)	32.81 (19.27–54.92)	26.61 (15.75–44.28)	0.657 (0.435–0.925)	16.92 (11.22–23.85)	19.16 (12.59–27.09)
Both sexes						
High SDI	0.086 (0.070–0.107)	8.96 (7.31–11.22)	15.06 (12.36–19.20)	0.070 (0.055–0.092)	6.96 (5.40–9.05)	12.58 (9.74–16.50)
High-middle SDI	0.136 (0.103–0.164)	10.05 (7.06–12.06)	15.55 (11.59–18.63)	0.103 (0.077–0.130)	7.19 (5.37–9.09)	12.03 (8.86–15.14)
Middle SDI	0.329 (0.263–0.434)	14.93 (11.95–19.70)	17.53 (13.98–23.37)	0.257 (0.204–0.324)	10.71 (8.51–13.50)	13.88 (10.97–17.80)
Low-middle SDI	0.374 (0.251–0.542)	23.87 (16.02–34.57)	20.97 (14.11–30.32)	0.318 (0.231–0.425)	18.05 (13.07–24.08)	18.41 (13.32–24.55)
Low SDI	0.333 (0.181–0.542)	30.97 (20.04–60.13)	21.23 (11.82–33.95)	0.345 (0.216–0.525)	30.58 (19.11–46.52)	19.98 (12.67–29.92)
Female						
High SDI	0.032 (0.023–0.043)	6.65 (4.88–8.96)	11.19 (8.09–15.45)	0.027 (0.020–0.056)	5.33 (3.90–7.23)	9.61 (6.91–13.33)
High-middle SDI	0.057 (0.039–0.073)	0.837 (5.66–10.70)	13.23 (8.71–17.14)	0.043 (0.030–0.057)	6.08 (4.22–7.96)	10.41 (7.03–13.69)
Middle SDI	0.131 (0.099–0.183)	11.97 (9.11–16.78)	14.37 (10.88–20.51)	0.104 (0.079–0.146)	8.79 (6.61–12.28)	11.64 (8.76–16.33)
Low-middle SDI	0.148 (0.083–0.259)	19.11 (10.67–33.43)	17.16 (9.58–30.00)	0.127 (0.076–0.191)	14.46 (8.70–21.80)	15.09 (8.98–22.92)
Low SDI	0.131 (0.064–0.257)	29.48 (14.40–57.48)	17.38 (8.81–33.12)	0.135 (0.077–0.224)	24.05 (13.63–39.84)	16.15 (9.28–26.34)
Male						
High SDI	0.053 (0.042–0.072)	11.30 (8.76–15.26)	18.73 (14.31–25.72)	0.044 (0.031–0.059)	8.61 (6.22–11.74)	15.40 (11.06–11.73)
High-middle SDI	0.079 (0.053–0.098)	11.75 (7.92–14.45)	17.66 (11.61–21.77)	0.059 (0.040–0.076)	8.31 (5.61–10.66)	13.52 (8.81–17.44)
Middle SDI	0.199 (0.151–0.282)	17.83 (13.52–25.33)	20.43 (25.42–29.19)	0.151 (0.114–0.202)	12.60 (9.47–16.74)	15.95 (11.97–21.17)
Low-middle SDI	0.226 (0.125–0.341)	28.53 (15.80–42.99)	24.53 (13.62–36.88)	0.192 (0.112–0.281)	21.61 (12.46–31.69)	21.52 (12.46–31.69)
Low SDI	0.201 (0.095–0.382)	44.36 (20.89–84.284)	24.90 (12.09–46.56)	0.210 (0.110–0.366)	37.07 (19.47–64.69)	21.51 (12.46–31.69)

In addition, there were apparent differences at gender level. In 2019, there were comparable incidence rates in female and male UCAs, while the death rate of MUCAs (male urogenital congenital anomalies) was much higher than the rate of FUCAs (female urogenital congenital anomalies). Similarly, there was 11.35 (95% UI 7.83–15.66) per 100,000 of DALYs rate worldwide attributable to FUCAs in 2019, compared with 16.92 (95% UI 11.22–23.85) per 100 000 attributable to MUCAs (Table 1, Supplementary Tables 2, 3).

### Variation in UCA burden at regional, national levels and SDI region with sex differences

**Figure 1:**
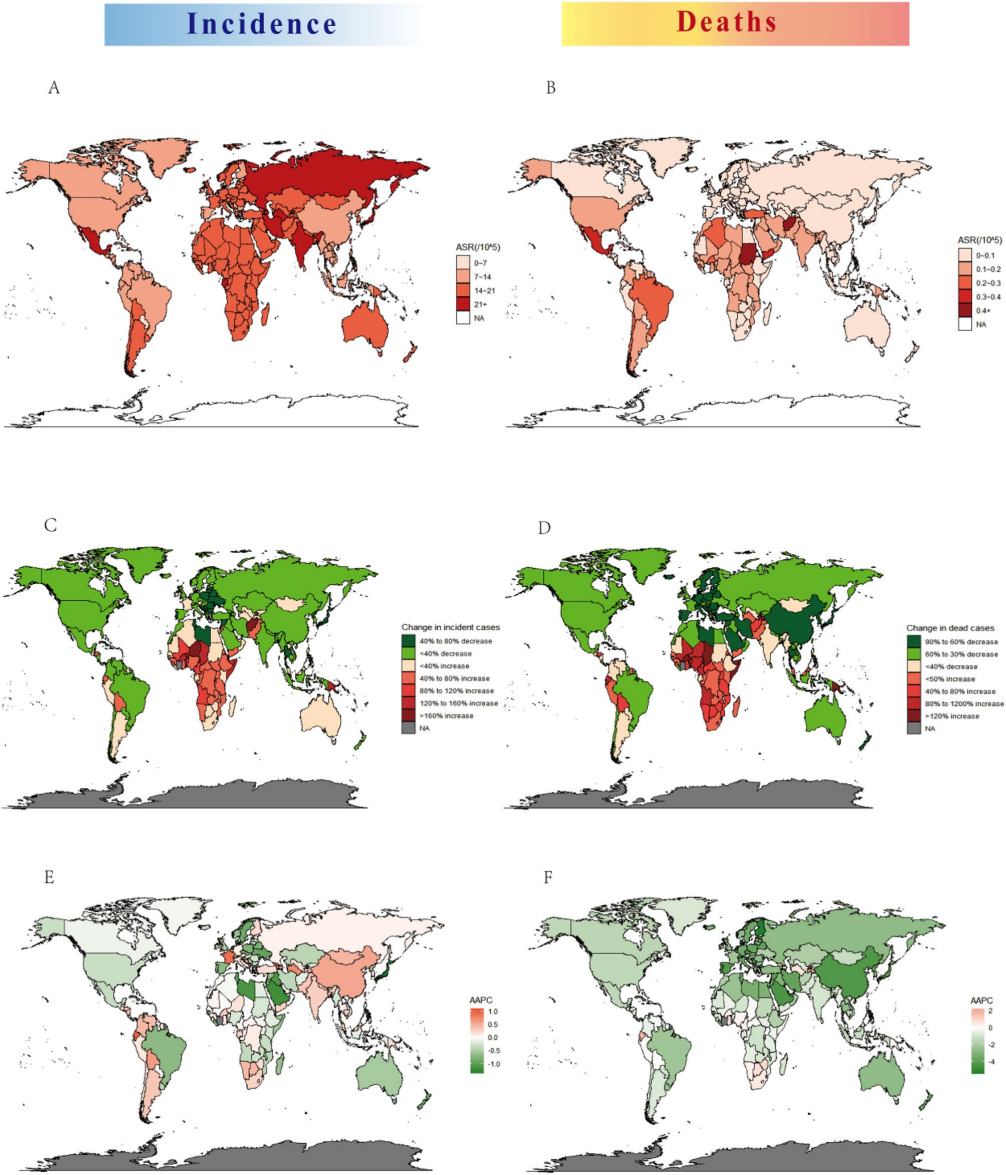
The global disease burden of urogenital congenital anomalies in 204 countries and territories. (**A**) ASIR in 2019; (**B**) ASDR in 2019; (**C**) change in incidence from 1990 to 2019; (**D**) change in cases of death from 1990 to 2019; (**E**) AAPC of ASIR from 1990 to 2019; (**F**) AAPC of ASDR from 1990 to 2019. *ASIR* age-standardized incidence rate, *ASDR* age-standardized deaths rate, *AAPC* average annual percentage change.

We displayed the geographic distribution of ASDR and ASIR for UCAs in 2019 in Fig. 1. Among the 21 GBD regions, high-income Asia Pacific, and Eastern Europe were the top 2 regions in ASPR (age-standardized prevalence rate) of UCAs in 2019 (162.11 and 150.99 per 100,000 population). Alarmingly, tropical Latin America showed not only the highest ASPR but also the second highest ASDR in 2019. It was also shown that East Asia held the lowest ASDR in 2019 and presented the largest decrease in ASDR from 1990 to 2019 (EAPC =  − 4.97; 95% UI − 5.24 to 0.41) (Supplementary Table 4).

The countries with the largest populations, including India, China, Nigeria, Pakistan, the United States of America, and Mexico had the more incident cases, prevalent cases, death cases, and higher DALYs rates in 2019, and all four indicators of India were significantly high. From 1990 to 2019, China was estimated to have a large increase in the ASIR but with a large decrease in the ASDR (AAPC of ASIR = 0.55; AAPC of ASDR =  − 4.3). The average annual percentage change(AAPC) of ASIRs exceeding 0 was found in nearly half of 204 countries and territories, such as El Salvador, Georgia, Ecuador, France, and Turkmenistan. Moreover, the AAPC of ASDR exceeding 0.1 was observed in 25 other countries and territories, such as Tajikistan, Ecuador, El Salvador, Georgia, and Turkmenistan (Fig. 1, Supplementary Figs. 1, 2).

When examining gender-based differences, the ASPR and ASDR distributions among male and female UCAs in various GBD regions were found to be similar. In 2019, East Asia exhibited the lowest ASDR for both FUCAs and MUCAs (as indicated in Supplementary Table 3). Notably, there was a significant disparity between FUCAs and MUCAs in terms of ASIR and the corresponding trends when analyzed at the geographic distribution level. For instance, in 2019, the ASIR in the United States of America decreased by 1.0 (95% UI − 1.1 to − 0.9) for FUCAs, while it increased to 0.2 (95% UI 0.2–0.3) for MUCAs (as shown in Supplementary Table 4).

### The influential factors for UCAs burden

In 2019, the age-standardized rates (ASRs) of deaths and DALYs were both highest in low-SDI regions, followed by low-middle-SDI regions, and lowest in high-middle-SDI regions and high-SDI regions. The ASIR was lowest in high SDI regions, with a significant decreasing from 1990 to 2019. Furthermore, it was observed that the ASRs of DALYs and deaths decreased across all SDI regions. Between 1990 and 2019, the ASIRs and ASDRs for MUCAs were consistently higher than those for FUCAs in all SDI regions. In 2019, the ASDRs and ASIRs for MUCAs were nearly ten times higher than those for FUCAs, and the gap in ASIRs between FUCAs and MUCAs widened. Additionally, in the high SDI region, while the ASDRs for FUCAs remained consistently low, the ASDRs for MUCAs significantly decreased from the highest in 1990 to nearly the lowest in 2019 over the past three decades (see Supplementary Table 4).

We further analyzed the relationship between the initial burden of ASIR and ASDR in 1990 and estimated annual percentage change (EAPC) values in 204 countries or territories. At the national level, there existed a slight negative correlation between the EAPC of ASIR or ASDR and initial ASIR in 1990, which was observed in both male and female cases. Figure 3 illustrates the observed correlation between regional, national age-standardized rates (ASRs) in 2019 with the anticipated levels based on Socio-Demographic Index (SDI) for each geographical location. Notably, a significant negative association was discovered between ASDRs (p < 0.0001) or ASRs (p < 0.0001) of DALYs and SDI, regardless of whether the analysis was conducted at the regional or national level. Over the period spanning from 1990 to 2019, the ASDR and ASR of DALYs demonstrated a discernible decline in the majority of GBD regions, with the exception of Central Asia, which remained relatively stable. Supplementary Figure 3 illustrates that the relationship between ASIRs in 2019 and SDI is complex. While the ASIR generally decreases as the SDI increases, it exhibits an increase at a certain point. These associations are consistent at gender levels (Supplementary Figs. 3–5).

**Figure 2:**
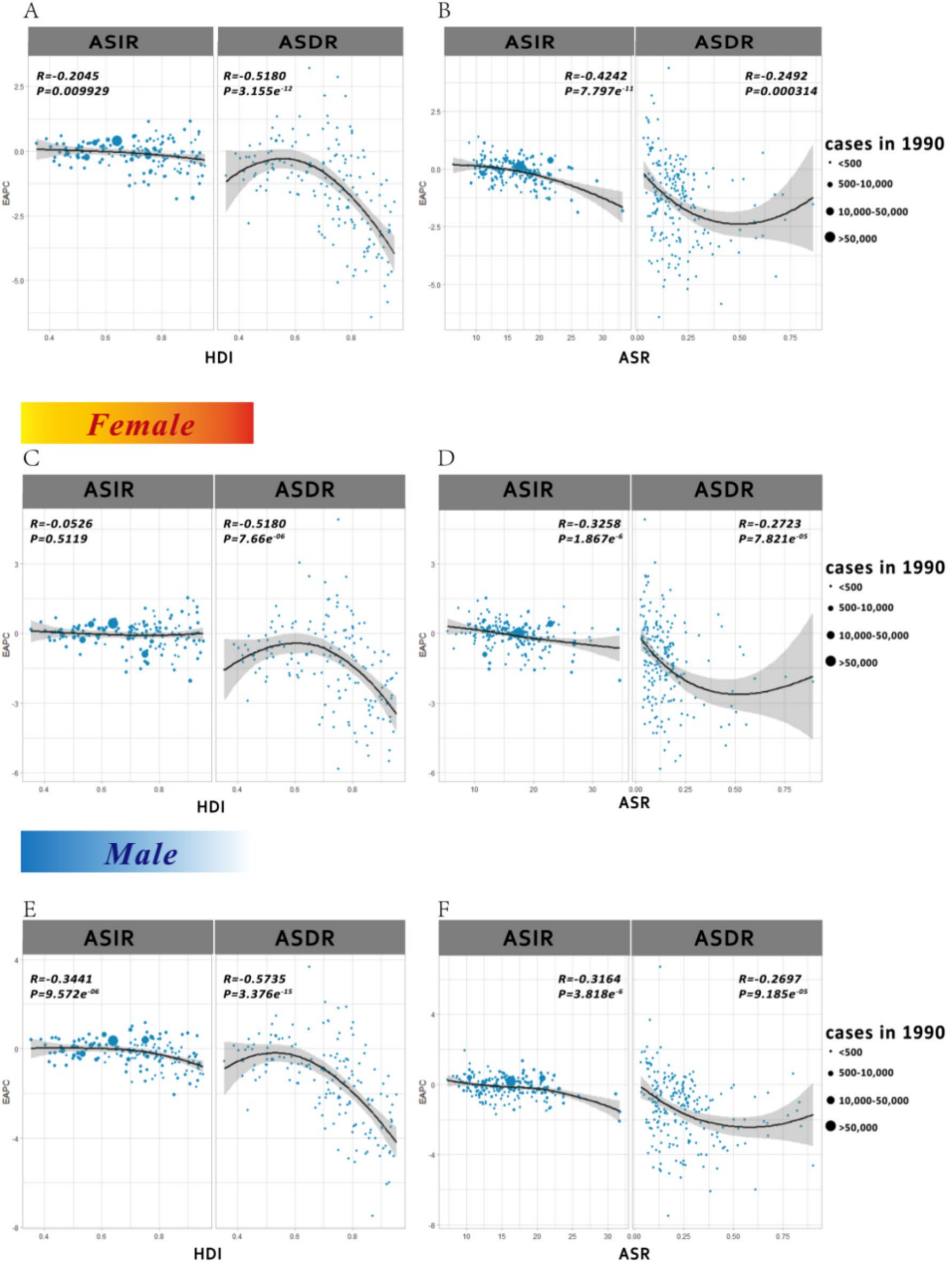
The influential factors for the EAPC of UCAs. (**A**) The correlation between the EAPC for ASIR and ASDR in 2019 and HDI in 2016; (**B**) the correlation between the EAPC for ASIR or ASDR and ASIR or ASDR in 1990. For female urogenital congenital anomalies, (**C**) the correlation between the EAPC for ASIR and ASDR in 2019 and HDI in 2016; (**D**) the correlation between the EAPC for ASIR or ASDR and ASIR or ASDR in 1990. For male urogenital congenital anomalies, (**E**) the correlation between the EAPC for ASIR and ASDR in 2019 and HDI in 2016; (**F**) the correlation between the EAPC for ASIR or ASDR and ASIR or ASDR in 1990.The circles represent countries that had available on HDI data. The size of the circle increased the number of cases. The q indices and p values presented were derived from Pearson’s correlation analysis. *ASR* age-standardized rate, *EAPC* estimated annual percentage change, *HDI* human development index.

Moreover, we found that the EAPCs in ASRs were negatively associated with the human development index (HDI) (in 2016). The ASR of UCAs in 1990 reflects the disease reservoir at baseline and the HDI in 2016 can serve as a surrogate for the level and availability of health care in each country. Countries with higher HDIs underwent a more rapid decrease in the ASR of UCAs from 1990 to 2016. However, this association was not found between EAPC in ASIR for female UCAs and HDI (Fig. 2).

### The potential environmental risk factors

In order to identify potential risk factors for UCAs, an analysis was conducted to examine the relationship between environmental exposures of GBD risk factors (as described in Supplementary Table 1) in 1990 and ASIRs or ASDRs of UCAs in 21 GBD regions. The results indicated a negative correlation between the summary exposure value (SEV) of low temperature in 1990 and ASDR in 2019, which was observed in both male and female cases. Conversely, the SEV of diet elements deficiency, unsafe water, and air pollution were positively associated with ASIR and ASDR of UCAs, although this association was not observed in male cases. Additionally, only the SEV of a diet low in seafood omega-3 fatty acids was found to be positively related to ASIR of UCAs in males. Positive associations were observed between the SEV of unsafe water, unsafe sanitation, child and maternal malnutrition, and a diet low in vegetables with ASDRs in both males and females. However, the relationship between SEV of dietary element deficiencies in 1990 and ASDRs in 2019 varied by gender. Specifically, positive correlations were found between SEV of vitamin A deficiency or zinc deficiency and ASDRs in males, but not in females. Conversely, SEV of iron deficiency was positively associated with ASDRs in FUCAs.

## Discussion

Based on the GBD 2019, this study showed the very first insight into the worldwide disease burden of UCAs from 1990 to 2019. Globally, there were 10,200 all-ages death cases of UCAs and incidence rate of 8.38 per 1000 live births in 2019, with around a total of 6.28 million individuals living with such anomalies. A study on Chinese children showed congenital anomalies of the kidney and urinary tract (CAKUTs) in 489 out of 26,989 (1.67%)^16^. Evaluating CAKUTs in infants in Italy for a period of 18 years proposed an incidence of 0.96% among infants^17^. A study estimated that the global CAKUTs are highly prevalent, accounting for 20–30% of prenatally detected malformations^18,19^. These studies, together with the present study, confirm that UCAs affects a large number of patients worldwide and is a serious public health condition threatening human health.

In the current study, the worldwide prevalence, incidence, mortality, DALYs, sex differences, and potential risk factors of UCAs have been comprehensively analyzed. The results reveal that there is a slight upward trend of age-standardized rate of prevalence and incidence for UCAs, whereas the age-standardized deaths rate and disability-adjusted life years (DALYs) both show relatively decreasing trends over the past 30 years globally. The reduction in ASDR for UCAs can be primarily attributed to a significant decline in death cases among live births, which may be partly attributed to advancements in medical therapies. Additionally, the observed increase in ASIR can be attributed to more precise detection in antenatal diagnosis. Owing to improvement in antenatal diagnostic technologies, most UCAs can be identified and classified at an early stage of pregnancy using ultrasonography, radiation, computed tomography (CT), magnetic resonance imaging (MRI), and new genetic approaches^20,21^. These findings confirm that the burden of UCAs is undergoing significant transformation.

The estimates of UCAs burden varied across regions and countries. Our data showed that only the high SDI regions showed decreasing trends in the all ASRs of prevalence, incidence, mortality, and DALYs from 1990 to 2019. A possible explanation is that in high SDI regions, much of the decrease can be attributed to the serious problem of aging and lower birth rates, as well as the development of technologies^22^ and public health care^23–26^. Such inconsistencies in regions could also be explained by differences in malformation inclusion criteria and study population. However, the additional influence of other non-etiologic factors and etiologic factors (including genetic and environmental risk factors) cannot be excluded. Strikingly, the ASPR and ASIR of UCAs in all high-income Asia Pacific countries were remarkably high among 204 countries in 2019, though they are all high-income level regions. These results therefore need to be interpreted with caution^4,27^.

**Figure 3:**
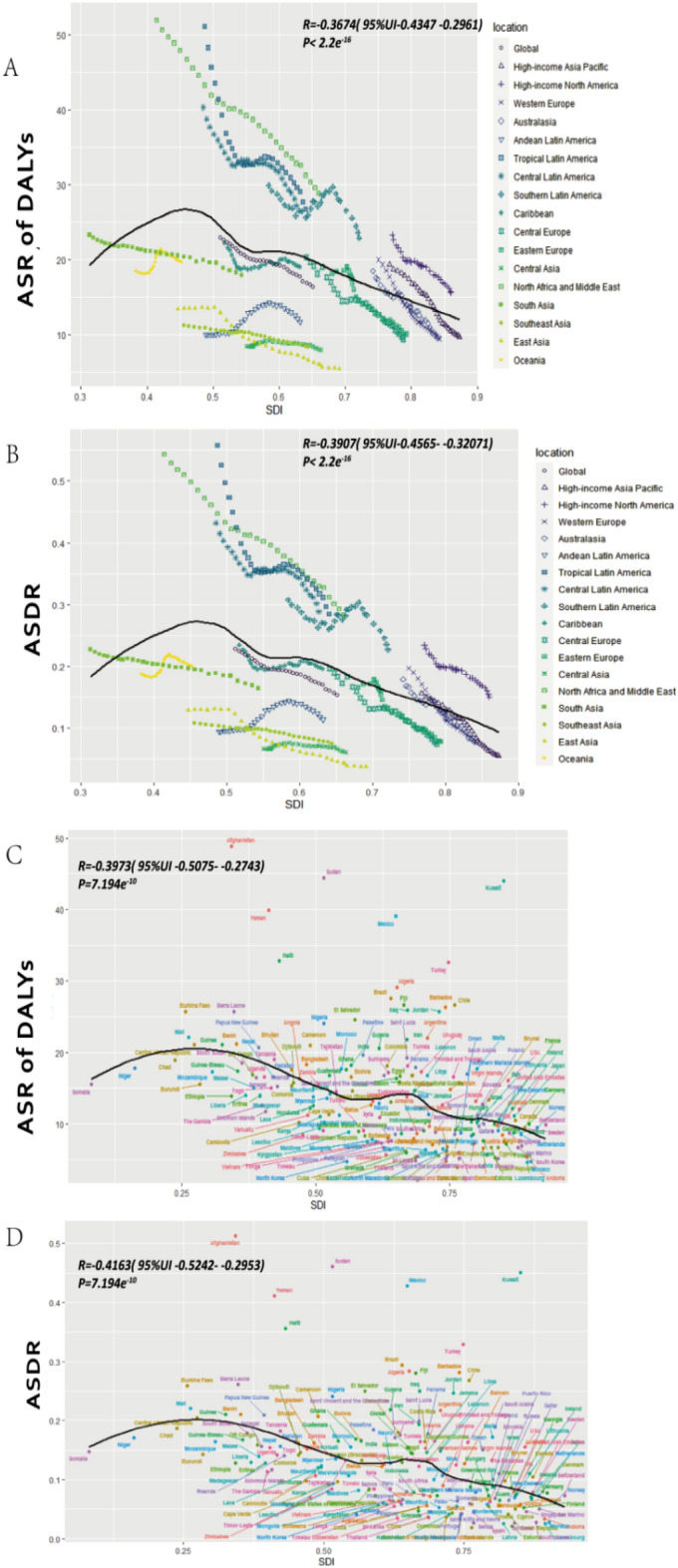
The correlation between SDI and ASRs in 2019 at regional and national levels. (**A**) ASRs of DALYs and (**B**) ASDRs of Urogenital Congenital Anomalies in 21 GBD regions from 1990 to 2019. (**C**) ASRs of DALYs and (**D**) ASDRs of UCAs in 204 countries and territories in 2019. Expected values based on SDI and disease rates in all locations are shown as the black line. *SDI* socio-demographic index, *DALYs* disability-adjusted life years.

This study further found positive associations between socioeconomic status and the burden of UCAs which has not been previously reported. Based on our analysis, we have reported that the decrease in ASR of DALYs and ASDR attributable to UCAs is correlated with the increasing SDI in 2019, as depicted in Fig. 3 and Table 1. Furthermore, when we incorporated another indicator, the Human Development Index (HDI), we observed consistent findings. The estimated annual percent changes (EAPCs) in age-standardized rates (ASRs) were inversely associated with the HDI in 2016, indicating that countries with higher HDIs experienced a more significant decline in ASRs for UCAs from 1990 to 2016. In developing countries, more medical research and more detailed prevention and intervention strategies should be involved^28,29^.

Remarkably, our research found that the UCA burden was significantly higher in men than in women, with the ratio of men to women in terms of deaths rates, prevalence rates, and DALYs being approximately 1.5:1. From 1990 to 2019, in all level SDI regions, the ASIRs and ASDRs for UCAs in men were higher than those in women. Furthermore, this sex differences in the burden of UCA showed a slight enlarging trend because the decrease in ASIRs and ASDRs was more pronounced in females. A distinct male predominance in cases of CAKUTs has been documented in multiple studies^30,31^. Studies conducted in China found a high proportion of CAKUTs in male newborns^32,33^, and reports from Saudi Arabia also indicated that approximately 66% of CAKUT cases were male^34^. However, the reason for this sex differences is not well understood^23,35^. Our data sources encompass a diverse range of congenital urogenital diseases, and the specific target diseases are not explicitly identified. Given these limitations, it becomes challenging to discuss and investigate the potential causes for sex differences in UCAs. To address this concern, it is advisable to focus on specific diseases or a narrower subset of UCAs. By narrowing down the scope, more focused investigations could be conducted.

The etiology of UCA is extremely complex and uncertain and calls for further research. However, the majority of such malformations lack a clear genetic origin or familial inheritance pattern^18,36^. Since maternal environment can have significant impact on kidney development^37–39^, we have made a tentative exploration on the potential environmental risk factors. Our results found a negative correlation between the exposure of low temperature and ASDRs of UCAs, which is never be reported before. Additionally, positive associations were identified between the exposure of unsafe water, unsafe sanitation, child and maternal malnutrition, a diet low in vegetables, and ASDRs of UCAs. Notably, these environmental risk factors were all linked to the level of national development, especially the unsafe water and unsafe sanitation, according to WHO. Our analysis also showed that the correlation between the exposure of diet element deficiencies and burden of UCAs varied at gender level, which also help us to explore sex differences in the disease burden of UCAs. There were positive correlations between the SEV of vitamin A deficiency or zinc deficiency and ASDRs of MUCAs, while the SEV of Iron deficiency was positive related to ASDR of FUCAs.

The first documented cases of severe renal aplasia/hypoplasia, horseshoe kidney, and ureteral anomalies resulting from vitamin A deficiency date back 70 years^40^. After that the association of maternal vitamin A deficiency with nephron reduction was studied on a rat model and pregnant women^41–43^. A recent study observed significant overlaps between vitamin A and the CAKUT gene sets^44^. Notably, vitamin A signaling depends on the availability of retinoic acid (RAs) and retinoic acid receptors (Rars) including Rara and Rarb^45^. Retinoic acid signaling plays a crucial role in the development and maturation of both the upper and lower urinary tract, partly via its activation of Ret gene expression, whose reduce leads to impaired branching morphogenesis^46–48^. In Rara−/− Rarb2−/− male rats, instead of joining the bladder, dilated ureters were connected directly to the vas deferens, which was also dilated, while Rara−/− Rarb2−/− females showed the distal ureters joining the uterus or vagina, instead of the bladder^47^. However, the sex differences and potential lethality resulting from these anomalies have not been thoroughly examined. It is also noteworthy that exposure to a vitamin A/retinoic acid deficient diet during gestation has been found to cause a distinctive range of malformations (dysgenesis of seminiferous tubules, seminal vesicles, epididymis, prostate, genital tubercle/hypospadias, and cryptorchidism) in the male rat fetus^49–51^.

A study reported that the fetuses from zinc-deficient females contained less zinc than did their controls, and almost all of the full-term fetuses produced under such conditions showed gross congenital malformations encompassing a wide variety of organ systems, including urogenital defects^52^. Another study found that zinc-deficient male offspring at 6 days showed decreased glomerular filtration areas, remodeling of renal arteries, greater number of renal apoptotic cells, while female offspring would appear to be less sensitive to zinc deficiency^53^. As regards the association between UCAs and iron deficiency, there was no previous research to support this.

These findings may help to predict potential factors of sex differences detected in UCAs burden. However, further clinical trials and research should be conducted to verify.

## Limitations of this study

There are still several limitations that should be noted in our study, although GBD contributors have improved the data and methods used to estimate the burden of diseases based on all available data sources. First, GBD estimates of the disease burden are constructed on the basis of mathematical models using limited sources of data from epidemiological surveys and are subject to a degree of deviation from actual data, particularly in some severely aging countries and in some underdeveloped regions where prior information is extremely scarce. Second, to a certain extent, the burden of UCAs could be underestimated due to the high likelihood of undiagnosed and untreated UCAs in newborns in developing countries. Third, GBD data are collected from different databases and institutional findings, making it difficult to avoid having differently defined disease categories and diagnoses, which can lead to heterogeneity in the data and affect the consistency of burden estimates.

Fourth, this study lacks a further estimate of the risk factors leading to the burden of UCAs and the genetic and environmental factors that contribute to the huge sex differences. More research will focus on this aspect in the future.

## Conclusions and policy implications

In summary, UCAs is a major public health problem globally, with great variation among countries. Although the age standardized prevalence, incidence, deaths and DALYs of UCAs showed a decreasing trend over the past thirty years, its burden remains high, with people in low income countries and men particularly at risk. Increasing the awareness of population and policy makers about UCAs and its risk factors, together with providing preventive and curative interventions for people living with UCAs, is highly recommended for reducing the future burden of this condition.

### Supplementary Information

Supplementary Information.

## Data Availability

Data was acquired from the Global Health Data Exchange (https://vizhub.healthdata.org/gbd-results/), and GBD (https://www.healthdata.org/).

## Abbreviations

UCAs: Urogenital congenital anomalies
GBD 2019: Global Burden of Disease, Injury, and Risk Factor Study 2019
UI: Uncertainty interval
SDI: Socio-Demographic Index
ASR: Age-standardized rate
DALYs: Disability-adjusted life years
GHDx: Global health data exchange
AAPC: Average annual percentage change
EAPC: Estimated annual percentage change
CI: Confidence interval
ASPR: Age-standardized prevalence rate
HDI: Human development index
ASDR: Age-standardized deaths rate
ASIR: Age-standardized incidence rate
SEV: Summary exposure value
FUCAs: Female urogenital congenital anomalies
MUCAs: Male urogenital congenital anomalies
