# Supplementary material for: Sex difference and risk factors in burden of urogenital congenital anomalies from 1990 to 2019
Source: Sci Rep. 2023 Aug 22;13:13656. doi: 10.1038/s41598-023-40939-3 (PMC10444850; doi:10.1038/s41598-023-40939-3)

# Supplementary Table 1 GBD 2017 risk factor hierarchy and accompanying exposure definitions

|  | **Risk factors** | | | **Exposure definition** | **Theoretical minimum risk exposure level** |  |
| --- | --- | --- | --- | --- | --- | --- |
| **0** | **All** | | | **..** | **..** |  |
| **1** | **Environmental and occupational risks** | | | **..** | **..** |  |
| **2** |  | **Unsafe water, sanitation, and hand-washing** | | **..** | **..** |  |
| 3 |  | Unsafe water source | | Proportion of individuals with access to different water sources (unimproved, improved except piped, or piped water supply) and reported use of household water treatment methods (boiling or filtering, chlorinating or solar filtering, or no treatment) | All individuals have access to water from a piped water supply that is also boiled or filtered before drinking |  |
| 3 |  | Unsafe sanitation | | Proportion of individuals with access to different sanitation facilities (unimproved, improved except sewer, or sewer connection) | All individuals have access to toilets with sewer connection |  |
| 3 |  | No access to hand-washing facility | | Proportion of individuals with access to handwashing facility with soap, water, and wash station | All individuals have access to handwashing facility with soap, water, and wash station |  |
| **2** | **Air pollution** | | | **..** | **..** |  |
| 3 | Particulate matter pollution | | | .. | .. |  |
| 4 |  | Ambient particulate matter pollution | | Annual average daily exposure to outdoor air concentrations of particulate matter with an aerodynamic diameter of ≤2·5 μm (PM2·5), measured in μg/m3 | Joint theoretical minimum risk exposure level for both household and ambient particulate matter pollution is a uniform distribution between 2·4 and 5·9 μg/m3, with burden attributed proportionally between household and particulate matter pollution on the basis of source of PM2·5 exposure in excess of theoretical minimum risk exposure level |  |
| 4 |  | Household air pollution from solid fuels | | Individual exposure to PM2·5 due to use of solid cooking fuel | See ambient particulate matter pollution |  |
| 3 | Ambient ozone pollution | | | Seasonal (6-month period with highest ozone) 8-h daily maximum ozone concentrations, measured in ppb | Uniform distribution between 29·1 and 35·7 ppb |  |
| **2** | **Other environmental risks** | | | **..** | **..** |  |
| 3 | Residential radon | | | Average daily exposure to indoor air radon levels measured in becquerels (radon disintegrations per second) per cubic metre (Bq/m3) | 10 Bq/m3, corresponding to the outdoor concentration of radon |  |
| 3 | Lead exposure | | | Blood lead levels in μg/dL of blood, bone lead levels in μg/g of bone | 2 μg/dL, corresponding to lead levels in pre-industrial humans as natural sources of lead prevent the feasibility of zero exposure |  |
| **2** |  | **Occupational risks** | | **..** | **..** |  |
| 3 |  | Occupational carcinogens | | .. | .. |  |
| 4 |  |  | Occupational exposure to asbestos | Proportion of the population with cumulative lifetime exposure to occupational asbestos | No occupational exposure to asbestos | |
| 4 |  |  | Occupational exposure to arsenic | Proportion of the population ever exposed to arsenic at work or through their occupation | No occupational exposure to arsenic | |
| 4 |  |  | Occupational exposure to benzene | Proportion of the population ever exposed to benzene at work or through their occupation | No occupational exposure to benzene | |
| 4 |  |  | Occupational exposure to beryllium | Proportion of the population ever exposed to beryllium at work or through their occupation | No occupational exposure to beryllium | |
| 4 |  |  | Occupational exposure to cadmium | Proportion of the population ever exposed to cadmium at work or through their occupation | No occupational exposure to cadmium | |
| 4 |  |  | Occupational exposure to chromium | Proportion of the population ever exposed to chromium at work or through their occupation | No occupational exposure to chromium | |
| 4 |  |  | Occupational exposure to diesel engine exhaust | Proportion of the population ever exposed to diesel engine exhaust at work or through their occupation | No occupational exposure to diesel engine exhaust | |
| 4 |  |  | Occupational exposure to formaldehyde | Proportion of the population ever exposed to formaldehyde at work or through their occupation | No occupational exposure to formaldehyde | |
| 4 |  |  | Occupational exposure to nickel | Proportion of the population ever exposed to nickel at work or through their occupation | No occupational exposure to nickel | |
| 4 |  |  | Occupational exposure to polycyclic aromatic hydrocarbons | Proportion of the population ever exposed to polycyclic aromatic hydrocarbons at work or through their occupation | No occupational exposure to polycyclic aromatic hydrocarbons | |
| 4 |  |  | Occupational exposure to silica | Proportion of the population ever exposed to silica at work or through their occupation | No occupational exposure to silica | |
| 4 |  |  | Occupational exposure to sulphuric acid | Proportion of the population ever exposed to sulphuric acid at work or through their occupation | No occupational exposure to sulphuric acid | |
| 4 |  |  | Occupational exposure to trichloroethylene | Proportion of the population ever exposed to trichloroethylene at work or through their occupation | No occupational exposure to trichloroethylene | |
| 3 |  | Occupational asthmagens | | Proportion of the population currently exposed to asthmagens at work or through their occupation | Background asthmagen exposures |  |
| 3 |  | Occupational particulate matter, gases, and fumes | | Proportion of the population ever exposed to particulates, gases, or fumes at work or through their occupation | No occupational exposure to particulates, gases, or fumes |  |
| 3 |  | Occupational noise | | Proportion of the population ever exposed to noise greater than 85 decibels at work or through their occupation | Background noise exposure |  |
| 3 |  | Occupational injuries | | Proportion of the population at risk to injuries related to work or through their occupation | The rate of injury deaths per 100 000 person-years is zero |  |
| 3 |  | Occupational ergonomic factors | | Proportion of the population who are exposed to ergonomic risk factors for low back pain at work or through their occupation | All individuals have the ergonomic factors of clerical and related workers |  |
| **1** | **Behavioural risks** | | | **..** | **..** |  |
| **2** |  | **Child and maternal malnutrition** | | **..** | **..** |  |
| 3 |  | Suboptimal breastfeeding | | .. | .. |  |
| 4 |  |  | Non-exclusive breastfeeding | Proportion of children younger than 6 months who receive predominant, partial, or no breastfeeding | All children are exclusively breastfed for first 6 months of life | |
| 4 |  |  | Discontinued breastfeeding | Proportion of children aged 6–23 months who do not receive any breast milk | All children continue to receive breast milk until 2 years of age | |
| 3 |  | Child growth failure | | .. | .. |  |
| 4 |  |  | Child underweight | Proportion of children ≥3 SDs, 2–3 SDs, and 1–2 SDs lower than the WHO 2006 standard weight-for-age curve | All children are <1 SD below the WHO 2006 standard weight-for-age curve | |
| 4 |  |  | Child wasting | Proportion of children ≥3 SDs, 2–3 SDs, and 1–2 SDs lower than the WHO 2006 standard weight-for-length curve | All children are <1 SD below the WHO 2006 standard weight-for-height curve | |
| 4 |  |  | Child stunting | Proportion of children ≥3 SDs, 2–3 SDs, and 1–2 SDs lower than the WHO 2006 standard height-for-age curve | All children are <1 SD below the WHO 2006 standard height-for-age curve | |
| 3 |  | Low birthweight and short gestation | | .. | .. |  |
| 4 |  |  | Low birthweight for gestation | Proportion of births occurring in 2-week gestational age categories from [0–24) weeks to [40–42) weeks, for each 500-g birthweight category starting from [0–500) g to [4000–4500) g[*](https://www.ncbi.nlm.nih.gov/pmc/articles/PMC6227755/table/tbl1/?report=objectonly#tbl1fn1) | 500-g birthweight category with lowest risk within each gestational age category | |
| 4 |  |  | Short gestation for birthweight | Proportion of births occurring in 500-g birthweight categories from [0–500) g to [4000–4500) g, for each 2-week gestational age category starting from [0–24) weeks to [40–42) weeks[*](https://www.ncbi.nlm.nih.gov/pmc/articles/PMC6227755/table/tbl1/?report=objectonly#tbl1fn1) | 2-week gestational age category with lowest risk within each birthweight category | |
| 3 |  | Iron deficiency | | Peripheral blood haemoglobin concentration in g/L for all iron-responsive causes | Counterfactual haemoglobin concentration in the absence of iron deficiency in g/L for all iron-responsive causes |  |
| 3 |  | Vitamin A deficiency | | Proportion of children aged 0–5 years with serum retinol concentration <0·7 μmol/L | No childhood vitamin A deficiency |  |
| 3 |  | Zinc deficiency | | Proportion of the population with inadequate zinc intake versus loss | No inadequate zinc intake |  |
| **2** |  | **Tobacco** | | **..** | **..** |  |
| 3 |  | Smoking | | Prevalence of current use of any smoked tobacco product and prevalence of former use of any smoked tobacco product; among current smokers, cigarette equivalents smoked per smoker per day and cumulative pack-years of exposure; among former smokers, number of years since quitting | All individuals are lifelong non-smokers |  |
| 3 |  | Chewing tobacco | | Current use of any chewing tobacco product | All individuals are lifelong non-users of chewing tobacco products |  |
| 3 |  | Second-hand smoke | | Average daily exposure to air particulate matter from second-hand smoke with an aerodynamic diameter smaller than 2·5 μg, measured in μg/m3, among non-smokers | No second-hand smoke exposure |  |
| **2** |  | **Alcohol use** | | **Average daily alcohol consumption of pure alcohol (measured in g per day) in current drinkers who had consumed alcohol during the past 12 months** | **Estimated distribution 0–10 g per day** |  |
| **2** |  | **Drug use** | | **Proportion of the population dependent upon opioids, cannabis, cocaine, or amphetamines; proportion of the population who have ever injected drugs** | **No drug use** |  |
| **2** |  | **Dietary risks** | | **..** | **..** |  |
| 3 |  | Diet low in fruits | | Average daily consumption of fruits (fresh, frozen, cooked, canned, or dried, excluding fruit juices and salted or pickled fruits) | Consumption of fruit 200–300 g per day |  |
| 3 |  | Diet low in vegetables | | Average daily consumption of vegetables (fresh, frozen, cooked, canned, or dried, excluding legumes and salted or pickled vegetables, juices, nuts and seeds, and starchy vegetables such as potatoes or corn) | Consumption of vegetables 290–430 g per day |  |
| 3 |  | Diet low in legumes | | Average daily consumption of legumes (fresh, frozen, cooked, canned, or dried legumes) | Consumption of legumes 50–70 g per day |  |
| 3 |  | Diet low in whole grains | | Average daily consumption of whole grains (bran, germ, and endosperm in their natural proportion) from breakfast cereals, bread, rice, pasta, biscuits, muffins, tortillas, pancakes, and other sources | Consumption of whole grains 100–150 g per day |  |
| 3 |  | Diet low in nuts and seeds | | Average daily consumption of nut and seed foods | Consumption of nuts and seeds 16–25 g per day |  |
| 3 |  | Diet low in milk | | Average daily consumption of milk, including non-fat, low-fat, and full-fat milk, excluding soy milk and other plant derivatives | Consumption of milk 350–520 g per day |  |
| 3 |  | Diet high in red meat | | Average daily consumption of red meat (beef, pork, lamb, and goat but excluding poultry, fish, eggs, and all processed meats) | Consumption of red meat 18–27 g per day |  |
| 3 |  | Diet high in processed meat | | Average daily consumption of meat preserved by smoking, curing, salting, or addition of chemical preservatives | Consumption of processed meat 0–4 g per day |  |
| 3 |  | Diet high in sugar-sweetened beverages | | Average daily consumption of beverages with ≥50 kcal per 226·8 g serving, including carbonated beverages, sodas, energy drinks, fruit drinks, but excluding 100% fruit and vegetable juices | Consumption of sugar-sweetened beverages 0–5 g per day |  |
| 3 |  | Diet low in fibre | | Average daily intake of fibre from all sources including fruits, vegetables, grains, legumes, and pulses | Consumption of fibre 19–28 g per day |  |
| 3 |  | Diet low in calcium | | Average daily intake of calcium from all sources, including milk, yogurt, and cheese | Consumption of calcium 1·0–1·5 g per day |  |
| 3 |  | Diet low in seafood omega 3 fatty acids | | Average daily intake of eicosapentaenoic acid and docosahexaenoic acid | Consumption of seafood omega 3 fatty acids 200–300 mg per day |  |
| 3 |  | Diet low in polyunsaturated fatty acids | | Average daily intake of omega 6 fatty acids from all sources, mainly liquid vegetable oils, including soybean oil, corn oil, and safflower oil | Consumption of polyunsaturated fatty acids as 9–13% of total daily energy |  |
| 3 |  | Diet high in trans fatty acids | | Average daily intake of trans fat from all sources, mainly partially hydrogenated vegetable oils and ruminant products | Consumption of trans fatty acids as 0–1% of total daily energy |  |
| 3 |  | Diet high in sodium | | 24-h urinary sodium measured in g per day | 24-h urinary sodium 1–5 g per day |  |
| **2** |  | **Intimate partner violence** | | **Proportion of the population who have ever experienced one or more acts of physical or sexual violence by a present or former intimate partner since age 15 years** | **No intimate partner violence** |  |
| **2** |  | **Childhood maltreatment** | | **..** | **..** |  |
| 3 |  | Childhood sexual abuse | | Proportion of the population ever having had the experience of intercourse or other contact abuse (ie, fondling and other sexual touching) when aged 15 years or younger, and the perpetrator or partner was more than 5 years older than the victim | No childhood sexual abuse |  |
| 3 |  | Bullying victimisation | | Proportion of population attending school who have been exposed to bullying victimisation within the past year | No bullying victimisation |  |
| **2** |  | **Unsafe sex** | | **Proportion of the population with exposure to sexual encounters that convey the risk of disease** | **No exposure to disease-causing pathogen through sex** |  |
| **2** |  | **Low physical activity** | | **Average weekly physical activity at work, home, transport-related and recreational measured by MET min per week** | **All adults experience 3000–4500 MET min per week** |  |
| **1** | **Metabolic risks** | | | **..** | **..** |  |
| **2** |  | **High fasting plasma glucose** | | **Serum fasting plasma glucose measured in mmol/L** | **4·8–5·4 mmol/L** |  |
| **2** |  | **High low-density lipoprotein cholesterol** | | **Serum low-density lipoprotein, measured in mmol/L** | **0·7–1·3 mmol/L** |  |
| **2** |  | **High systolic blood pressure** | | **Systolic blood pressure, measured in mm Hg** | **110–115 mm Hg** |  |
| **2** |  | **High body-mass index** | | **Body-mass index, measured in kg/m2** | **20–25 kg/m2** |  |
| **2** |  | **Low bone mineral density** | | **Standardised mean bone mineral density values measured by dual x-ray absorptiometry at the femoral neck in g/cm2** | **99th percentile of NHANES 1988–2014 by age and sex** |  |
| **2** |  | **Impaired kidney function** | | **Proportion of the population with ACR >30 mg/g or GFR <60 mL/min/1·73 m2, excluding end-stage renal disease** | **GFR >60 mL/min/1·73 m2 and ACR <30 mg/g** |  |

ACR=albumin-to-creatine ratio. GBD=Global Burden of Diseases, Injuries, and Risk Factors Study. GFR=glomerular filtration rate. MET=metabolic equivalent. NHANES=National Health and Nutrition Examination Survey. PM2·5=particulate matter with an aerodynamic diameter smaller than 2·5 μm, measured in μm/m3. ppb=parts per billion.

*In numbered intervals, square brackets indicate included endpoints and round brackets indicate excluded endpoints.

Supplementary Table 2: Global prevalence, incidence, deaths, YLLs, YLDs and DALYs in number and age-standardised rates for both sexes combined, females, and males, in 2019, with percentage change between 2010 and 2019.YLLs, years of life lost; YLDs, years lived with disability; DALYs, disability adjusted life years.

|  | Prevalence | | Incidence | | Deaths | | YLLs | | YLDs | | DALYs | |
| --- | --- | --- | --- | --- | --- | --- | --- | --- | --- | --- | --- | --- |
|  | Cases  (millions) | Rate  (per 100 000) | Cases  (millions) | Rate  (per 100 000) | Deaths  (millions) | Rate  (per 100 000) | YLLs  (millions) | Rate  (per 100 000) | YLDs  (millions) | Rate  (per 100 000) | DALYs  (millions) | Rate  (per 100 000) |
| 2019 | | | | | | | | | | | | |
| Both Sexes | 6.28  (4.98- 7.72) | 89.2  (70.5- 109.7) | 1.13  (0.795- 1.62) | 17.5  (12.3- 25.0) | 0.0102  (0.00755- 0.0134) | 0.2  (0.1- 0.2) | 0.868  (0.630- 1.15) | 13.2  (9.6- 17.6) | 0.227  (0.129- 0.363) | 3.2  (1.8- 5.2) | 1.09  (0.832- 1.41) | 16.4  (12.4- 21.0) |
| Females | 3.17  (2.53- 3.88) | 92.4  (73.4- 113.4) | 0.559  (0.392- 0.790) | 17.9  (12.5- 25.3) | 0.00392  (0.00242- 0.00571) | 0.1  (0.1- 0.2) | 0.329  (0.203- 0.489) | 10.3  (6.4- 15.4) | 0.109  (0.0617- 0.174) | 3.2  (1.8- 5.1) | 0.438  (0.302- 0.604) | 13.5  (9.2- 18.7) |
| Males | 3.11  (2.45- 3.83) | 86.1  (67.4- 106.3) | 0.576  (0.405- 0.833) | 17.2  (12.1- 24.9) | 0.00630  (0.00378- 0.00906) | 0.2  (0.1- 0.3) | 0.538  (0.321- 0.783) | 15.9  (9.5- 23.2) | 0.118  (0.0668- 0.189) | 3.3  (1.8- 5.2) | 0.657  (0.435- 0.925) | 19.2  (12.6- 27.1) |
| Percentage change 2010-2019 | | | | | | | | | | | | |
| Both Sexes | 5.1%  (2.1- 8.2) | 1.7%  (–1.3- 4.7) | 5.4%  (0.8- 10.3) | 6.8%  (2.1- 11.7) | –15.9%  (–29.3- 3.5) | –16.2%  (–29.6- 3.1) | –16.9%  (–30.4- 2.9) | –16.6%  (–30.2- 3.1) | 5.3%  (1.2- 9.3) | 1.8%  (–2.4- 5.7) | –13.1%  (–25.1- 3.4) | –13.6%  (–25.6- 3.0) |
| Females | 5.7%  (2.8- 8.7) | 2.2%  (–0.6- 5.2) | 5.4%  (1.0- 10.9) | 6.6%  (2.3- 12.3) | –15.9%  (–32.2- 3.6) | –16.6%  (–32.6- 2.8) | –17.1%  (–33.4- 2.7) | –17.1%  (–33.4- 2.6) | 6.0%  (1.6- 10.2) | 2.5%  (–1.7- 6.9) | –12.4%  (–28.1- 2.8) | –13.2%  (–28.8- 2.0) |
| Males | 4.5%  (1.0- 8.0) | 1.1%  (–2.4- 4.6) | 5.5%  (0.3- 10.6) | 7.0%  (1.6- 12.1) | –15.9%  (–29.2- 5.6) | –15.9%  (–29.2- 5.8) | –16.7%  (–30.- 5.1) | –16.3%  (–29.7- 5.7) | 4.7%  (0.4- 9.1) | 1.2%  (–3.1- 5.6) | –13.5%  (–26.2- 5.0) | –13.7%  (–26.3- 4.9) |
| Numbers in parentheses are 95% uncertainty intervals. | | | | | | | | | | | | |

Supplementary Table 3 Top10 countries and territories in highest and lowest ASDR, ASIR, ASPR and age-standardized rates of DALYs of Urogenital Congenital Anomalies in 2019. Top10 countries and territories in highest and lowest ASDR, ASIR, ASPR and age-standardized rates of DALYs of Female and Male Urogenital Congenital Anomalies in 2019.ASIR, age-standardized incidence rate; ASDR, age-standardized deaths rate; ASPR, age-standardized prevalence rate; DALYs, disability adjusted life years.

| **Gender** |  | | | | | | | | | | |
| --- | --- | --- | --- | --- | --- | --- | --- | --- | --- | --- | --- |
| Both Sexes |  | | | | | | | | | | |
|  | **Top10 in highest ASDR** | |  | **Top10 in lowest ASDR** | |  | **Top10 in highest ASPR** | |  | **Top10 in lowest ASPR** | |
|  | Country | Region |  | Country | Region |  | Country | Region |  | Country | Region |
|  | Afghanistan | North Africa and Middle East |  | Cook Islands | Oceania |  | Japan | High‐income  Asia‐Pacific |  | Paraguay | Tropical Latin  America |
|  | Sudan | North Africa and Middle East |  | Estonia | Eastern Europe |  | Iran (Islamic Republic of) | North Africa and Middle East |  | Canada | High‐income North America |
|  | Kuwait | North Africa and  Middle East |  | Luxembourg | Western Europe |  | Singapore | High‐income  Asia‐Pacific |  | Greenland | High‐income North America |
|  | Mexico | Central Latin  America |  | Latvia | Eastern Europe |  | Russian Federation | Eastern Europe |  | Democratic People's Republic of Korea | East Asia |
|  | Yemen | North Africa and  Middle East |  | Serbia | Central Europe |  | Brunei Darussalam | High‐income  Asia‐Pacific |  | Brazil | Tropical Latin  America |
|  | Haiti | Caribbean |  | Slovenia | Central Europe |  | Republic of Korea | High‐income  Asia‐Pacific |  | Ecuador | Andean Latin  America |
|  | Turkey | North Africa and  Middle East |  | Monaco | Western Europe |  | Mexico | Central Latin  America |  | Peru | Andean Latin  America |
|  | Brazil | Tropical Latin  America |  | Republic of Korea | High‐income  Asia‐Pacific |  | Lithuania | Eastern Europe |  | Maldives | Southeast Asia |
|  | Algeria | North Africa and  Middle East |  | Antigua and Barbuda | Caribbean |  | Kuwait | North Africa and  Middle East |  | Thailand | Southeast Asia |
|  | Barbados | Caribbean |  | Andorra | Western Europe |  | Saudi Arabia | North Africa and  Middle East |  | Spain | Western Europe |
|  |  |  |  |  |  |  |  |  |  |  |  |
|  | **Top10 in highest ASIR** | |  | **Top10 in lowest ASIR** | |  | **Top10 in highest**  **ASR of DALYs** | |  | **Top10 in lowest**  **ASR of DALYs** | |
|  | Country | Region |  | Country | Region |  | Country | Region |  | Country | Region |
|  | Iran (Islamic Republic of) | North Africa and Middle East |  | Paraguay | Tropical Latin  America |  | Afghanistan | North Africa and Middle East |  | Cook Islands | Oceania |
|  | Russian Federation | Eastern Europe |  | Democratic People's Republic of Korea | East Asia |  | Sudan | North Africa and Middle East |  | Luxembourg | Western Europe |
|  | Kuwait | North Africa and  Middle East |  | Greenland | High‐income  North America |  | Kuwait | North Africa and  Middle East |  | Antigua and Barbuda | Caribbean |
|  | India | South Asia |  | Canada | High‐income  North America |  | Yemen | North Africa and  Middle East |  | Monaco | Western Europe |
|  | Singapore | High‐income  Asia‐Pacific |  | Saint Kitts and Nevis | Caribbean |  | Mexico | Central Latin  America |  | Democratic People's Republic of Korea | East Asia |
|  | Mexico | Central Latin  America |  | Dominica | Caribbean |  | Haiti | Caribbean |  | Thailand | Southeast Asia |
|  | Brunei Darussalam | High‐income  Asia‐Pacific |  | Antigua and Barbuda | Caribbean |  | Turkey | North Africa and  Middle East |  | Serbia | Central Europe |
|  | Azerbaijan | Central Asia |  | Sri Lanka | Southeast Asia |  | Algeria | North Africa and  Middle East |  | Slovenia | Central Europe |
|  | Japan | High‐income  Asia‐Pacific |  | Brazil | Tropical Latin  America |  | Brazil | Tropical Latin  America |  | Portugal | Western Europe |
|  | Gabon | Central sub‐  Saharan Africa |  | Spain | Western Europe |  | Fiji | Oceania |  | Estonia | Eastern Europe |
|  |  |  |  |  |  |  |  |  |  |  |  |
| **Gender** | **male** | | | | |  | **Female** | | | | |
| **ASPR** | **Top10 in highest ASPR** | |  | **Top10 in lowest ASPR** | |  | **Top10 in highest ASPR** | |  | **Top10 in lowest ASPR** | |
|  | Country | Region |  | Country | Region |  | Country | Region |  | Country | Region |
|  | Iran (Islamic Republic of) | North Africa and  Middle East |  | Brazil | Tropical Latin  America |  | Singapore | High‐income  Asia‐Pacific |  | Dominica | Caribbean |
|  | Japan | High‐income  Asia‐Pacific |  | Sri Lanka | Southeast Asia |  | Russian Federation | Eastern Europe |  | Bolivia (Plurinational State of) | Andean Latin  America |
|  | Bangladesh | South Asia |  | Timor-Leste | Southeast Asia |  | Lithuania | Eastern Europe |  | Peru | Andean Latin  America |
|  | Saudi Arabia | North Africa and  Middle East |  | Maldives | Southeast Asia |  | Norway | Western Europe |  | Brazil | Tropical Latin  America |
|  | Algeria | North Africa and  Middle East |  | Cambodia | Southeast Asia |  | Brunei Darussalam | High‐income  Asia‐Pacific |  | Canada | High‐income  North America |
|  | Kuwait | North Africa and  Middle East |  | Malaysia | Southeast Asia |  | Republic of Korea | High‐income  Asia‐Pacific |  | Ecuador | Andean Latin  America |
|  | Iraq | North Africa and  Middle East |  | Thailand | Southeast Asia |  | Latvia | Eastern Europe |  | Poland | Central Europe |
|  | India | South Asia |  | Paraguay | Tropical Latin  America |  | Republic of Moldova | Eastern Europe |  | Taiwan (Province of China) | East Asia |
|  | Egypt | North Africa and  Middle East |  | Greenland | High‐income  North America |  | Japan | High‐income  Asia‐Pacific |  | Democratic People's Republic of Korea | East Asia |
|  | Sudan | North Africa and  Middle East |  | Canada | High‐income  North America |  | Mexico | Central Latin  America |  | Paraguay | Tropical Latin  America |
|  |  |  |  |  |  |  |  |  |  |  |  |
| **ASIR** | **Top10 in highest ASIR** | |  | **Top10 in lowest ASIR** | |  | **Top10 in highest ASIR** | |  | **Top10 in lowest ASIR** | |
|  | Country | Region |  | Country | Region |  | Country | Region |  | Country | Region |
|  | Iran (Islamic Republic of) | North Africa and  Middle East |  | Paraguay | Tropical Latin  America |  | Russian Federation | Eastern Europe |  | Paraguay | Tropical Latin  America |
|  | Gabon | Central sub‐  Saharan Africa |  | Thailand | Southeast Asia |  | Singapore | High‐income  Asia‐Pacific |  | Democratic People's Republic of Korea | East Asia |
|  | Bangladesh | South Asia |  | Maldives | Southeast Asia |  | Azerbaijan | Central Asia |  | Taiwan (Province of China) | East Asia |
|  | India | South Asia |  | Spain | Western Europe |  | Georgia | Central Asia |  | Greenland | High‐income  North America |
|  | Central African Republic | Central sub‐  Saharan Africa |  | Sri Lanka | Southeast Asia |  | Lithuania | Eastern Europe |  | Canada | High‐income  North America |
|  | Kuwait | North Africa and  Middle East |  | Timor-Leste | Southeast Asia |  | Brunei Darussalam | High‐income  Asia‐Pacific |  | Brazil | Tropical Latin  America |
|  | Democratic Republic of the Congo | Central sub‐  Saharan Africa |  | Malaysia | Southeast Asia |  | Turkmenistan | Central Asia |  | United States of America | High‐income  North America |
|  | Japan | High‐income  Asia‐Pacific |  | Democratic People's Republic of Korea | East Asia |  | Republic of Moldova | Eastern Europe |  | Saint Kitts and Nevis | Caribbean |
|  | Taiwan (Province of China) | East Asia |  | Greenland | High‐income  North America |  | Mexico | Central Latin  America |  | Bahamas | Caribbean |
|  | Australia | Australasia |  | Monaco | Western Europe |  | Norway | Western Europe |  | Dominica | Caribbean |
|  | Saudi Arabia | North Africa and  Middle East |  | Croatia | Central Europe |  | Iran (Islamic Republic of) | North Africa and  Middle East |  | Ecuador | Andean Latin  America |
|  | Equatorial Guinea | Central sub‐  Saharan Africa |  | Mauritius | Southeast Asia |  | Kuwait | North Africa and  Middle East |  | Antigua and Barbuda | Caribbean |
|  | South Africa | Southern sub‐  Saharan Africa |  | Denmark | Western Europe |  | Armenia | Central Asia |  | Bermuda | Caribbean |
|  | New Zealand | Australasia |  | Antigua and Barbuda | Caribbean |  | India | South Asia |  | Jamaica | Caribbean |
|  | Algeria | North Africa and  Middle East |  | Seychelles | Southeast Asia |  | Tajikistan | Central Asia |  | Cuba | Caribbean |
|  | Nigeria | Western sub‐  Saharan Africa |  | Viet Nam | Southeast Asia |  | Mongolia | Central Asia |  | Samoa | Oceania |
|  | Albania | Central Europe |  | Belgium | Western Europe |  | Republic of Korea | High‐income  Asia‐Pacific |  | Portugal | Western Europe |
|  | Mexico | Central Latin  America |  | Slovenia | Central Europe |  | Japan | High‐income  Asia‐Pacific |  | Puerto Rico | Caribbean |
|  | Congo | Central sub‐  Saharan Africa |  | Honduras | Central Latin  America |  | Uzbekistan | Central Asia |  | Dominican Republic | Caribbean |
|  | Sierra Leone | Western sub‐  Saharan Africa |  | Dominica | Caribbean |  | Estonia | Eastern Europe |  | American Samoa | Caribbean |
| **ASDR** | **Top10 in highest ASDR** | |  | **Top10 in lowest ASDR** | |  | **Top10 in highest ASDR** | |  | **Top10 in lowest ASDR** | |
|  | Country | Region |  | Country | Region |  | Country | Region |  | Country | Region |
|  | Afghanistan | North Africa and  Middle East |  | Cook Islands | Oceania |  | Afghanistan | North Africa and  Middle East |  | Cook Islands | Oceania |
|  | Kuwait | North Africa and  Middle East |  | Barbuda | Caribbean |  | Barbados | Caribbean |  | Estonia | Eastern Europe |
|  | Sudan | North Africa and  Middle East |  | Estonia | Eastern Europe |  | Mexico | Central Latin  America |  | Republic of Korea | High‐income Asia‐Pacific |
|  | Mexico | Central Latin  America |  | Republic of Korea | High‐income Asia‐Pacific |  | Sudan | North Africa and  Middle East |  | Luxembourg | Western Europe |
|  | Yemen | North Africa and  Middle East |  | Luxembourg | Western Europe |  | Yemen | North Africa and  Middle East |  | Latvia | Eastern Europe |
|  | Haiti | Caribbean |  | Latvia | Eastern Europe |  | Kuwait | North Africa and  Middle East |  | Monaco | Western Europe |
|  | Turkey | North Africa and  Middle East |  | Monaco | Western Europe |  | Haiti | Caribbean |  | Serbia | Central Europe |
|  | Brazil | Tropical Latin  America |  | Portugal | Western Europe |  | Turkey | North Africa and  Middle East |  | Thailand | Southeast Asia |
|  | Sierra Leone | Western sub‐  Saharan Africa |  | Serbia | Central Europe |  | Fiji | Oceania |  | Tonga | Oceania |
|  | Burkina Faso | Western sub‐  Saharan Africa |  | Slovenia | Central Europe |  | Brazil | Tropical LatinAmerica |  | Uzbekistan | Central Asia |
|  |  |  |  |  |  |  |  |  |  |  |  |
| **ASR of**  **DALYs** | **Top10 in highest**  **ASR of DALYs** | |  | **Top10 in lowest**  **ASR of DALYs** | |  | **Top10 in larges**  **ASR of DALYs** | |  | **Top10 in lowest**  **ASR of DALYs** | |
|  | Country | Region |  | Country | Region |  | Country | Region |  | Country | Region |
|  | Afghanistan | North Africa and  Middle East |  | Cook Islands | Oceania |  | Afghanistan | North Africa and  Middle East |  | Cook Islands | Oceania |
|  | Kuwait | North Africa and  Middle East |  | Luxembourg | Western Europe |  | Sudan | North Africa and  Middle East |  | Democratic People's Republic of Korea | East Asia |
|  | Sudan | North Africa and  Middle East |  | Antigua and Barbuda | Caribbean |  | Mexico | Central Latin  America |  | Thailand | Southeast Asia |
|  | Yemen | North Africa and  Middle East |  | Estonia | Eastern Europe |  | Barbados | Caribbean |  | Mauritius | Southeast Asia |
|  | Mexico | Central Latin  America |  | Monaco | Western Europe |  | Yemen | North Africa and  Middle East |  | Bahamas | Caribbean |
|  | Haiti | Caribbean |  | Portugal | Western Europe |  | Kuwait | North Africa and  Middle East |  | Antigua and Barbuda | Caribbean |
|  | Turkey | Central Latin  America |  | Slovenia | Central Europe |  | Turkey | Central Latin  America |  | Cuba | Caribbean |
|  | Algeria | Central Latin  America |  | Latvia | Eastern Europe |  | Haiti | Caribbean |  | Monaco | Western Europe |
|  | Brazil | Tropical Latin  America |  | Uzbekistan | Central Asia |  | Fiji | Oceania |  | Luxembourg | Western Europe |
|  | Sierra Leone | Western sub‐  Saharan Africa |  | Serbia | Central Europe |  | Algeria | Central Latin  America |  | Spain | Western Europe |
|  |  |  |  |  |  |  |  |  |  |  |  |
| **Incidence**  **cases** | **Top10 in highest increase** | |  | **Top10 in highest decrease** | |  | **Top10 in highest increase** | |  | **Top10 in highest decrease** | |
|  | Country | Region |  | Country | Region |  | Country | Region |  | Country | Region |
|  | Afghanistan | North Africa and  Middle East |  | Puerto Rico | Caribbean |  | Niger | Western sub‐  Saharan Africa |  | Puerto Rico | Caribbean |
|  | Niger | Western sub‐  Saharan Africa |  | Republic of Moldova | Eastern Europe |  | Afghanistan | North Africa and  Middle East |  | Syria | North Africa and  Middle East |
|  | Chad | Western sub‐  Saharan Africa |  | Lithuania | Eastern Europe |  | Somalia | Eastern sub‐  Saharan Africa |  | Northern Mariana Islands | Oceania |
|  | Papua New Guinea | Oceania |  | Libya | North Africa and  Middle East |  | Chad | Western sub‐  Saharan Africa |  | Libya | North Africa and  Middle East |
|  | Qatar | Western sub‐  Saharan Africa |  | Syrian Arab Republic | North Africa and  Middle East |  | Benin | Western sub‐  Saharan Africa |  | Democratic People's Republic of Korea | East Asia |
|  | Somalia | Eastern sub‐  Saharan Africa |  | Democratic People's Republic of Korea | High‐income  Asia‐Pacific |  | Burkina Faso | Western sub‐  Saharan Africa |  | Moldova | Eastern Europe |
|  | Burkina Faso | Western sub‐  Saharan Africa |  | Ukraine | Eastern Europe |  | Mali | Western sub‐  Saharan Africa |  | Bosnia and Herzegovina | Central Europe |
|  | Mali | Western sub‐  Saharan Africa |  | Bosnia and Herzegovina | Central Europe |  | Papua New Guinea | Oceania |  | Lithuania | Eastern Europe |
|  | Benin | Western sub‐  Saharan Africa |  | Albania | Central Europe |  | Qatar | Western sub‐  Saharan Africa |  | Ukraine | Eastern Europe |
|  | Angola | Central sub‐  Saharan Africa |  | Northern Mariana Islands | Oceania |  | Cameroon | Western sub‐  Saharan Africa |  | Japan | High‐income  Asia‐Pacific |
| **Deaths cases** | **Top10 in highest increase** | |  | **Top10 in highest decrease** | |  | **Top10 in highest increase** | |  | **Top10 in highest decrease** | |
|  | Country | Region |  | Country | Region |  | Country | Region |  | Country | Region |
|  | Tajikistan | Central Asia |  | Estonia | Eastern Europe |  | Ecuador | Andean Latin  America |  | Syria | North Africa and  Middle East |
|  | Chad | Western sub-Saharan Africa |  | Finland | Western Europe |  | Somalia | Eastern sub‐  Saharan Africa |  | Puerto Rico | Caribbean |
|  | Somalia | Eastern  Sub-Saharan Africa |  | Syria | North Africa and  Middle East |  | Papua New Guinea | Oceania |  | Montenegro | Central Europe |
|  | Burkina Faso | Western  Sub-Saharan  Africa |  | Puerto Rico | Caribbean |  | Honduras | Central Latin  America |  | China | East Asia |
|  | Niger | Western  Sub-Saharan  Africa |  | Denmark | Western Europe |  | Cameroon | Western sub‐  Saharan Africa |  | Tunisia | North Africa and  Middle East |
|  | Angola | Central  Sub-Saharan  Africa |  | Lithuania | Eastern Europe |  | Chad | Western sub‐  Saharan Africa |  | Cook Islands | Oceania |
|  | Papua New Guinea | Oceania |  | Serbia | Central Europe |  | Burkina Faso | Western sub‐  Saharan Africa |  | Iran | North Africa and  Middle East |
|  | Cameroon | Western  Sub-Saharan  Africa |  | Czech Republic | Central Europe |  | Tajikistan | Central Asia |  | Denmark | Western Europe |
|  | Nigeria | Western  Sub-Saharan  Africa |  | Montenegro | Central Europe |  | Nigeria | Western sub‐  Saharan Africa |  | Serbia | Central Europe |
|  | Benin | Western  Sub-Saharan  Africa |  | Latvia | Eastern Europe |  | Mali | Western sub‐  Saharan Africa |  | Libya | North Africa and  Middle East |

Supplementary Table 4. Incidence and age-standardized incidence rate per 100 000 people for urogenital congenital anomalies, in 1990 and 2019, and their estimated annual percentage change from 1990 to 2019. No., number; ASIR, age-standardized incidence rate; ASDR, age-standardized deaths rate; UI, uncertainty interval; EAPC, estimated annual percentage change; CI, confidence interval.s

|  | 1990 | | |  | 2019 | | |  | 1990-2019 | |
| --- | --- | --- | --- | --- | --- | --- | --- | --- | --- | --- |
| Characteristics | Deaths cases  No. (95% UI) | ASDR  per 100 000  No. (95% UI) | ASIR  per 100 000  No. (95% UI) |  | Deaths cases  No. (95% UI) | ASDR  per 100 000  No. (95% UI) | ASIR  per 100 000  No. (95% UI) |  | EAPC in ASIR  No. (95% CI) | EAPC in ASDR  No. (95% CI) |
| Global | 14758.92 (8697.64-23388.21) | 0.23 (0.14-0.36) | 16.91 (11.88-24.11) |  | 10215.86 (7553-13440.77) | 0.15 (0.11-0.2) | 17.52 (12.28-25.05) |  | 0.09 (0.02-0.17) | -1.19 (-1.27-0.17) |
| SDI region |  |  |  |  |  |  |  |  |  |  |
| High SDI | 1281.82 (921.72-1891.99) | 0.22 (0.16-0.32) | 16.92 (12.42-22.86) |  | 601.75 (454.47-837.29) | 0.11 (0.08-0.16) | 14.83 (10.9-19.91) |  | -0.66 (-0.78--0.53) | -2.21 (-2.28--0.53) |
| High-middle SDI | 2236.63 (1465.45-3368.48) | 0.22 (0.14-0.33) | 16.05 (11.34-22.86) |  | 889.96 (633.88-1123.54) | 0.11 (0.07-0.14) | 16.9 (11.93-23.83) |  | 0.06 (-0.1-0.22) | -2.54 (-2.62-0.22) |
| Middle SDI | 4546.09 (2905.14-6976.24) | 0.22 (0.14-0.34) | 15.56 (10.95-22.09) |  | 2358.52 (1895.42-3069.68) | 0.13 (0.1-0.17) | 16.56 (11.57-23.27) |  | 0.11 (0-0.22) | -1.69 (-1.85-0.22) |
| Low-middle SDI | 4215.75 (2120.06-7764.77) | 0.24 (0.12-0.44) | 18.17 (12.56-26.26) |  | 2978.96 (2033.94-4111.52) | 0.17 (0.12-0.24) | 18.98 (13.27-27.53) |  | 0.21 (0.15-0.28) | -0.87 (-0.96-0.28) |
| Low SDI | 2470.43 (890.24-4936.65) | 0.23 (0.09-0.47) | 18.12 (12.49-26.33) |  | 3379.04 (1926.06-5463.74) | 0.19 (0.11-0.31) | 18.04 (12.54-26.18) |  | 0.04 (-0.02-0.11) | -0.43 (-0.51-0.11) |
| Gender |  |  |  |  |  |  |  |  |  |  |
| Male | 8975.96  (4873.10-15424.48) | 0.27(0.15-0.46) | 16.33(11.44-23.38) |  | 6295.65  (3777.04-9061.04) | 0.18(0.11-0.27) | 17.19(12.08-24.87) |  | 0.16 (0.09-0.24) | -1.07 (-1.16--0.97) |
| Female | 5782.96  (3350.26-11968.79) | 0.19(0.11-0.38) | 17.53(12.18-24.69) |  | 3920.21  (2417.03-5711.80) | 0.12(0.07-0.18) | 17.86(12.54-25.27) |  | 0.02 (-0.06-0.1) | -1.38 (-1.44--1.32) |
| GBD region |  |  |  |  |  |  |  |  |  |  |
| Tropical Latin America | 951.3 (397.50-1827.07) | 0.56 (0.23-1.07) | 12.34 (8.88-17.43) |  | 450.39 (272.38-596.2) | 0.28 (0.17-0.38) | 10.12 (7.35-14.1) |  | -0.76 (-0.94--0.57) | -1.58 (-1.89--0.57) |
| High-income North America | 523.08 (383.05-784.67) | 0.23 (0.17-0.35) | 12.43 (9.13-16.75) |  | 318.72 (217.23-435.06) | 0.15 (0.1-0.21) | 11.46 (8.52-15.19) |  | -0.48 (-0.59--0.37) | -1.18 (-1.3--0.37) |
| Caribbean | 99.27 (53.15-159.8) | 0.23 (0.13-0.38) | 12.55 (8.92-17.35) |  | 78.56 (39.61-137.53) | 0.2 (0.1-0.34) | 12.65 (8.99-17.67) |  | -0.16 (-0.24--0.09) | -0.23 (-0.4--0.09) |
| High-income Asia Pacific | 156.71 (97.08-191.77) | 0.16 (0.09-0.19) | 27.43 (19.41-39.02) |  | 46.51 (31.94-62.18) | 0.05 (0.03-0.08) | 21.1 (15.07-29.08) |  | -1.21 (-1.41--1) | -3.76 (-3.93--1) |
| Southeast Asia | 635.16 (327.72-1206.55) | 0.11 (0.06-0.2) | 12.81 (8.76-18.27) |  | 416.57 (312.96-563.26) | 0.08 (0.06-0.1) | 12.71 (8.63-18.33) |  | -0.14 (-0.19--0.09) | -1.13 (-1.24--0.09) |
| East Asia | 1557.5 (846.16-2293.52) | 0.13 (0.07-0.19) | 11 (7.64-15.3) |  | 364.39 (293.25-441.13) | 0.04 (0.03-0.05) | 12.88 (8.93-18.02) |  | -0.17 (-0.74-0.41) | -4.97 (-5.24-0.41) |
| Andean Latin America | 52 (32.5-106.91) | 0.09 (0.06-0.19) | 11.66 (8.3-16.51) |  | 71.98 (43.43-109) | 0.11 (0.07-0.17) | 13.22 (9.62-18.64) |  | 0.45 (0.37-0.53) | 1.43 (1.03-0.53) |
| Oceania | 18.99 (6.45-41.54) | 0.19 (0.07-0.4) | 13.73 (9.53-19.12) |  | 37.87 (13.94-82.78) | 0.2 (0.08-0.43) | 14.22 (9.73-20.33) |  | 0.26 (0.11-0.41) | 0.53 (0.33-0.41) |
| Western Europe | 462.39 (304.37-740.78) | 0.2 (0.13-0.32) | 15.14 (11.54-20.47) |  | 179.11 (132.98-282.85) | 0.08 (0.06-0.13) | 14.97 (11.12-20.05) |  | 0.05 (-0.05-0.15) | -3 (-3.15-0.15) |
| Central Europe | 165.85 (90.61-255.98) | 0.2 (0.11-0.31) | 17.75 (12.35-26.04) |  | 44.95 (27.03-60.87) | 0.07 (0.04-0.1) | 15.53 (10.84-22.9) |  | -0.46 (-0.68--0.24) | -3.03 (-3.19--0.24) |
| Southern Latin America | 154.49 (94.9-275.04) | 0.31 (0.19-0.55) | 15.25 (11.25-21.62) |  | 107.48 (51.93-152.38) | 0.23 (0.11-0.32) | 16.41 (11.77-23.24) |  | 0.41 (0.34-0.47) | -0.34 (-0.61-0.47) |
| Australasia | 28.24 (18.03-49.48) | 0.18 (0.11-0.32) | 19.85 (14.08-27.81) |  | 15.11 (10.69-25.72) | 0.08 (0.05-0.14) | 16.71 (11.91-23.1) |  | -0.62 (-0.67--0.57) | -2.49 (-2.69--0.57) |
| Eastern Sub-Saharan Africa | 746.46 (237.79-1711.56) | 0.19 (0.06-0.43) | 18.47 (12.65-26.66) |  | 927.5 (522.38-1576.53) | 0.14 (0.08-0.24) | 17.07 (11.89-24.6) |  | -0.1 (-0.18--0.02) | -0.55 (-0.72--0.02) |
| Central Asia | 60.62 (42.46-89.28) | 0.07 (0.05-0.1) | 16.74 (11.55-23.74) |  | 56 (34.52-76.67) | 0.06 (0.04-0.08) | 17.32 (11.91-24.58) |  | -0.08 (-0.19-0.03) | -0.05 (-0.28-0.03) |
| Central Latin America | 995.1 (436.99-2823.95) | 0.43 (0.2-1.2) | 17.94 (12.67-25.48) |  | 642.73 (450.53-1239.46) | 0.29 (0.2-0.58) | 17.37 (12.1-24.56) |  | 0.12 (-0.18-0.43) | -0.86 (-1.03-0.43) |
| Western Sub-Saharan Africa | 854.91 (229.47-2014.86) | 0.22 (0.06-0.5) | 19.16 (13.3-27.25) |  | 1624.25 (672.76-2915.25) | 0.22 (0.09-0.38) | 18.54 (12.86-26.54) |  | -0.05 (-0.12-0.02) | 0.14 (0.07-0.02) |
| Southern Sub-Saharan Africa | 41.67 (31.49-56.63) | 0.06 (0.05-0.08) | 17.37 (11.97-24.48) |  | 57 (38.24-79.89) | 0.07 (0.05-0.1) | 18.64 (12.95-26.06) |  | 0.48 (0.36-0.59) | 0.92 (0.8-0.59) |
| North Africa and Middle East | 2992.46 (1562.24-5542.2) | 0.54 (0.29-1.01) | 21.05 (14.71-31.27) |  | 1665.45 (1009.37-2265.6) | 0.28 (0.17-0.39) | 19.12 (13.32-28.15) |  | -0.42 (-0.54--0.29) | -2.06 (-2.13--0.29) |
| Central Sub-Saharan Africa | 247.43 (83.69-526.31) | 0.21 (0.07-0.44) | 19.24 (13.28-27.54) |  | 324.08 (180.6-538.45) | 0.16 (0.09-0.26) | 19.36 (13.05-28.23) |  | -0.2 (-0.34--0.06) | -0.61 (-0.74--0.06) |
| Eastern Sub-Saharan Africa | 3752.97 (1623.19-7044.87) | 0.23 (0.1-0.42) | 20.29 (13.91-29.64) |  | 2676.32 (1779.33-3924.37) | 0.16 (0.11-0.24) | 21.77 (14.99-31.87) |  | 0.33 (0.25-0.42) | -0.9 (-0.99-0.42) |
| Western Sub-Saharan Africa | 262.32 (194.82-537.16) | 0.17 (0.12-0.36) | 23.64 (16.31-34.24) |  | 110.91 (83.23-220.26) | 0.08 (0.05-0.18) | 23.09 (15.95-33.12) |  | -0.12 (-0.23--0.01) | -3.09 (-3.31--0.01) |

Supplementary Table 5 The correlation between SEV of risk factors and ASIR or ASDR in 2019. SEV, summary exposure value; ASIR, age-standardized incidence rate;ASDR, age-standardized Deaths rate. The Correlation coefficient and p values presented were derived from Pearson’s correlation analysis.

| **environmental exposure** | **p-value** | **Correlation coefficient** | **lower 95%** | **higher 95%** |
| --- | --- | --- | --- | --- |
|  | ASIR | | | |
| Unsafe water, sanitation, and handwashing | 0.044 | 0.4 | 0.012 | 0.68 |
| Air pollution | 0.044 | 0.398 | 0.012 | 0.68 |
| Low physical activity | 0.020 | -0.455 | -0.716 | -0.082 |
| Child and maternal malnutrition | 0.050 | 0.286 | -0.001 | 0.673 |
| Diet low in seafood omega-3 fatty acids | <0.001 | 0.636 | 0.329 | 0.821 |
| Diet low in calcium | 0.039 | 0.408 | 0.024 | 0.687 |
| Diet low in milk | 0.050 | 0.388 | 0.001 | 0.674 |
| Diet low in vegetable | 0.042 | 0.400 | 0.016 | 0.682 |
|  | ASDR | | | |
| Unsafe water, sanitation, and handwashing | 0.002 | 0.576 | 0.243 | 0.788 |
| Unsafe sanitation | 0.004 | 0.547 | 0.202 | 0.771 |
| Unsafe water source | 0.005 | 0.537 | 0.189 | 0.765 |
| No access to handwashing facility | 0.004 | 0.551 | 0.208 | 0.773 |
| Low temperature | 0.004 | -0.545 | -0.77 | -0.200 |
| Air pollution | 0.002 | 0.576 | 0.243 | 0.788 |
| Particulate matter pollution | 0.046 | 0.395 | 0.009 | 0.678 |
| Child and maternal malnutrition | 0.001 | 0.601 | 0.279 | 0.802 |
| Diet low in calcium | 0.006 | 0.527 | 0.176 | 0.760 |
| Diet low in vegetable | <0.001 | 0.709 | 0.443 | 0.860 |
| Iron deficiency | 0.001 | 0.604 | 0.283 | 0.803 |
| Vitamin A deficiency | 0.009 | 0.505 | 0.146 | 0.746 |
|  | ASIR of MUCAs | | | |
| Diet low in seafood omega-3 fatty acids | 0.011 | 0.490 | 0.127 | 0.737 |
|  | ASDR of MUCAs | | | |
| Unsafe water, sanitation, and handwashing | <0.001 | 0.633 | 0.326 | 0.820 |
| Unsafe sanitation | <0.001 | 0.609 | 0.290 | 0.806 |
| Unsafe water source | <0.001 | 0.623 | 0.311 | 0.814 |
| No access to handwashing facility | <0.001 | 0.638 | 0.333 | 0.822 |
| Low temperature | 0.004 | -0.549 | -0.772 | -0.205 |
| Air pollution | 0.011 | 0.489 | 0.126 | 0.737 |
| Particulate matter pollution | 0.011 | 0.492 | 0.129 | 0.738 |
| Child and maternal malnutrition | 0.016 | 0.468 | 0.099 | 0.724 |
| Diet low in seafood omega-3 fatty acids | 0.017 | 0.465 | 0.095 | 0.722 |
| Diet low in calcium | <0.001 | 0.639 | 0.335 | 0.823 |
| Diet low in milk | 0.034 | 0.417 | 0.035 | 0.692 |
| Diet low in vegetable | <0.001 | 0.757 | 0.524 | 0.885 |
| Vitamin A deficiency | 0.002 | 0.582 | 0.251 | 0.791 |
| Low bone mineral density | 0.030 | 0.426 | 0.046 | 0.698 |
| Zinc deficiency | 0.031 | 0.423 | 0.043 | 0.696 |
|  | ASIR of FUCAs | | | |
| Unsafe water, sanitation, and handwashing | 0.029 | 0.428 | 0.049 | 0.7 |
| Diet low in polyunsaturated fatty acids | 0.006 | 0.522 | 0.168 | 0.756 |
| Diet low in seafood omega-3 fatty acids | <0.001 | 0.575 | 0.241 | 0.787 |
|  | ASDR of FUCAs | | | |
| Unsafe water, sanitation, and handwashing | 0.030 | 0.427 | 0.047 | 0.699 |
| Unsafe sanitation | 0.034 | 0.417 | 0.035 | 0.693 |
| Low temperature | 0.008 | -0.506 | -0.747 | -0.147 |
| High fasting plasma glucose | 0.005 | 0.389 | 0.002 | 0.675 |
| Child and maternal malnutrition | 0.023 | 0.446 | 0.07 | 0.71 |
| Diet low in vegetable | 0.001 | 0.593 | 0.267 | 0.797 |
| Iron deficiency | 0.021 | 0.449 | 0.075 | 0.713 |

Supplementary Figure 1 The global disease burden of female urogenital congenital anomalies in 204 countries and territories. (A) ASIR in 2019; (B) ASDR in 2019; (C) Change of Incidence in Cases 2019 from 1990 to 2019; (D) Change of Deaths Cases from 1990 to 2019; (E) EAPC of ASIR from 1990 to 2019; (F)EAPC of ASDR from 1990 to 2019. (G) AAPC of ASIR from 1990 to 2019; (H)AAPC of ASDR from 1990 to 2019. ASIR, age-standardized incidence rate; ASDR, age-standardized deaths rate; EAPC, estimated annual percentage change. AAPC, average annual percentage change.


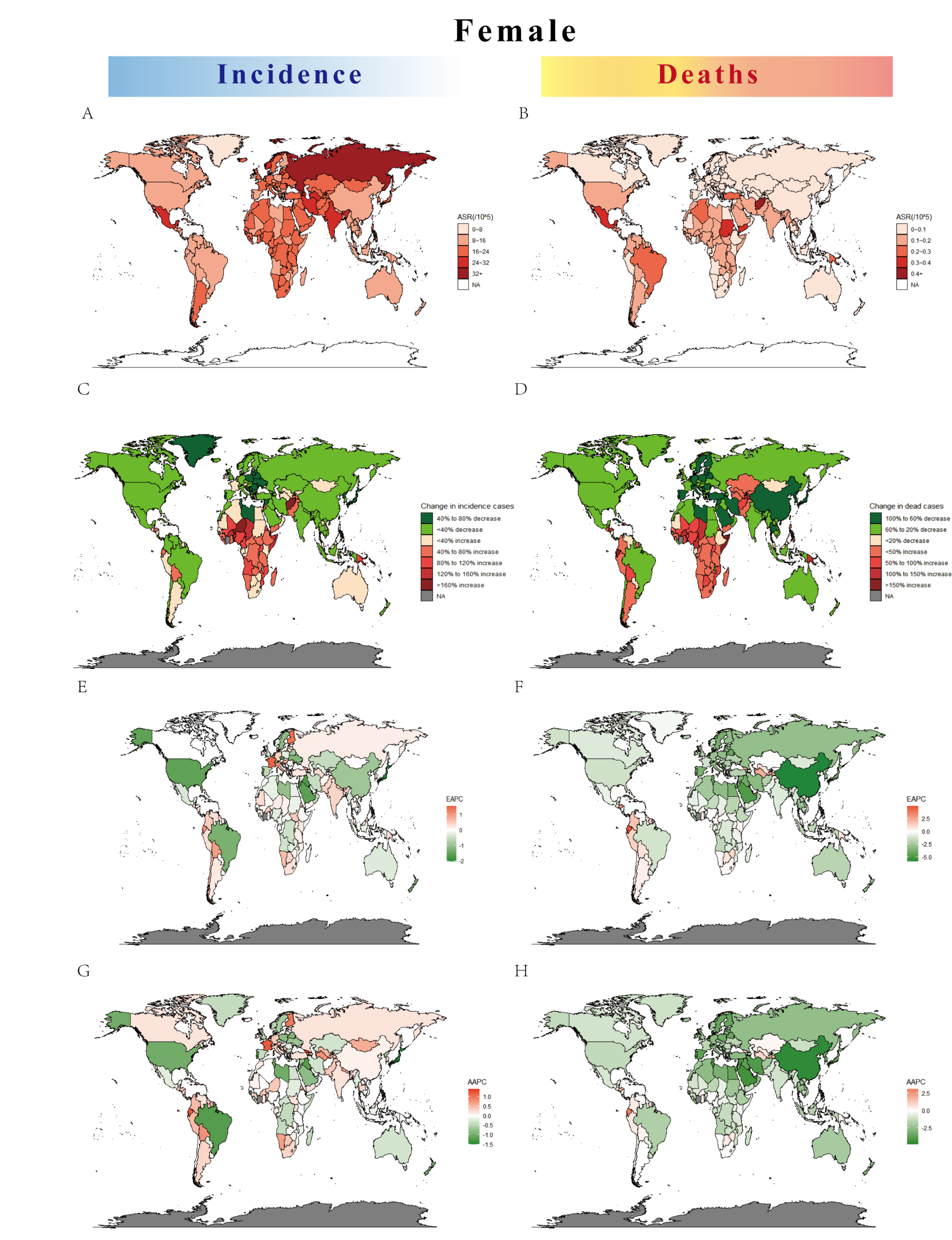


Supplementary Figure 2 The global disease burden of Male Urogenital Congenital Anomalies in 204 countries and territories. (A) ASIR in 2019; (B) ASDR in 2019; (C) Change of Incidence in Cases 2019 from 1990 to 2019; (D) Change of Deaths Cases from 1990 to 2019; (E) EAPC of ASIR from 1990 to 2019; (F)EAPC of ASDR from 1990 to 2019. (G) AAPC of ASIR from 1990 to 2019; (H)AAPC of ASDR from 1990 to 2019. ASIR, age-standardized incidence rate; ASDR, age-standardized deaths rate; EAPC, estimated annual percentage change. AAPC, average annual percentage change.


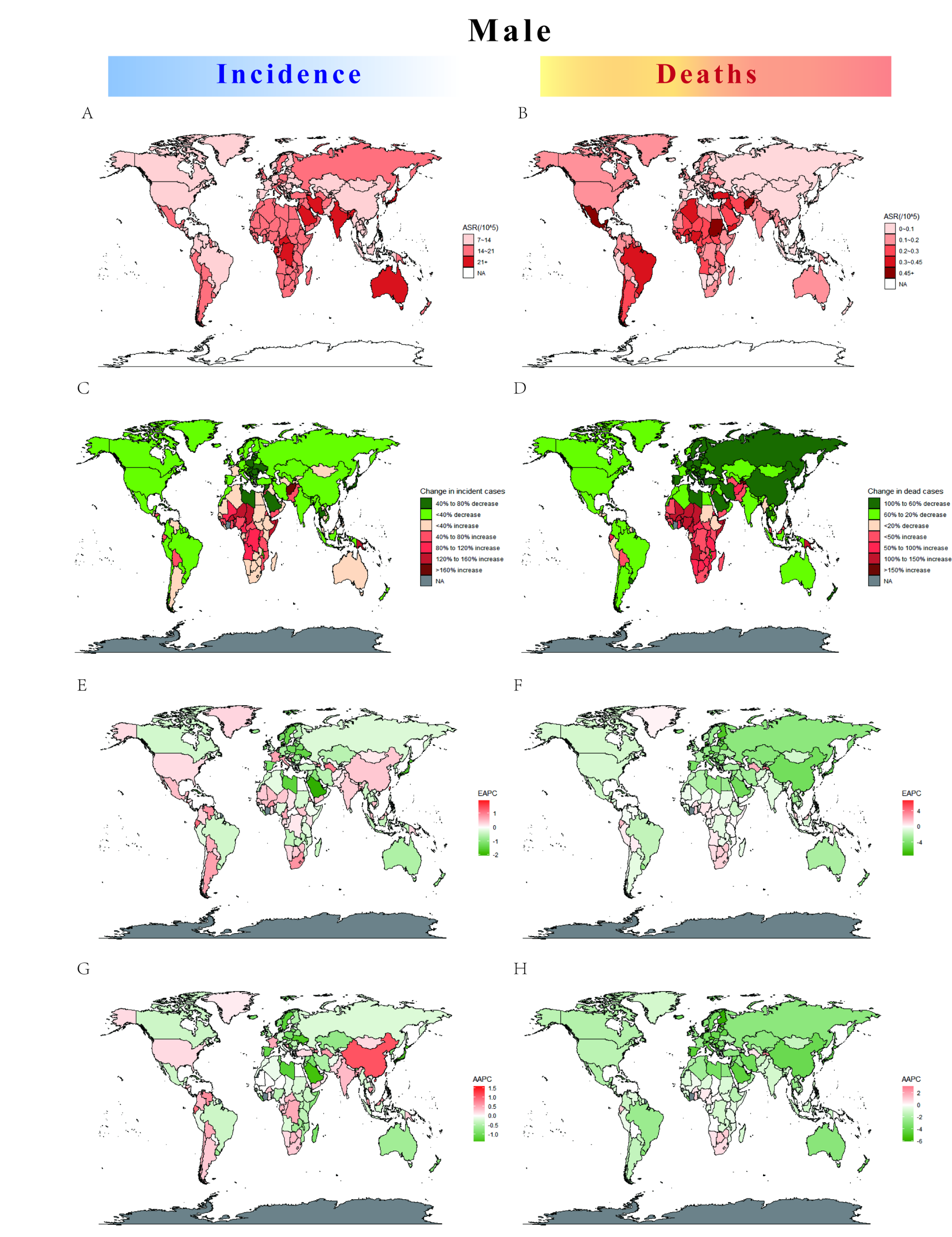


Supplementary Figure 3 The correlation between SDI and ASIRs of UCAs in 2019 at regional and national levels based on different gender grouping. Age-standardised incidence rates of Urogenital Congenital Anomalies (A) in 21 GBD regions from 1990 to 2019 and (B) in 204 countries and territories in 2019. Age-standardised incidence rates of Female Urogenital Congenital Anomalies (C) in 21 GBD regions from 1990 to 2019 and (D) in 204 countries and territories in 2019. Age-standardised incidence rates of Male Urogenital Congenital Anomalies (E) in 21 GBD regions from 1990 to 2019 and (F) in 204 countries and territories in 2019.Expected values based on SDI and disease rates in all locations are shown as the black line. GBD, Global Burden of Diseases. SDI, Socio-demographic Index.


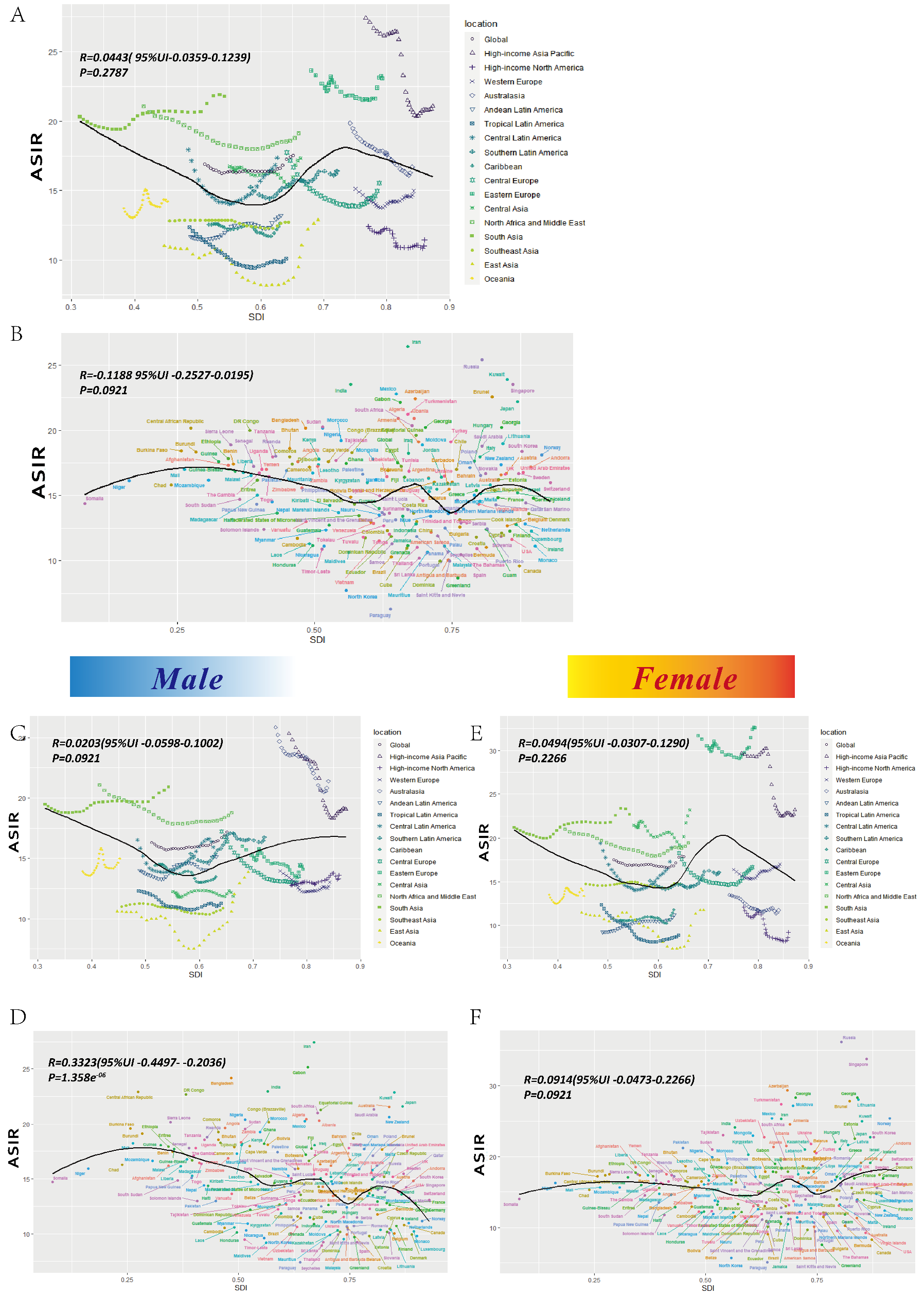


Supplementary Figure 4 The correlation between SDI and ASRs of FUCAs in 2019 at regional and national levels. (A)Age-standardized rates of DALYs and (B) age-standardized deaths rates of Female Urogenital Congenital Anomalies in 21 GBD regions from 1990 to 2019. (C)Age-standardized rates of DALYs and (D)age-standardized deaths rates of Urogenital Congenital Anomalies in 204 countries and territories in 2019. Expected values based on SDI and disease rates in all locations are shown as the black line.GBD,Global Burden of Diseases.SDI, Socio-demographic Index. DALYs, Disability Adjusted Life Years.


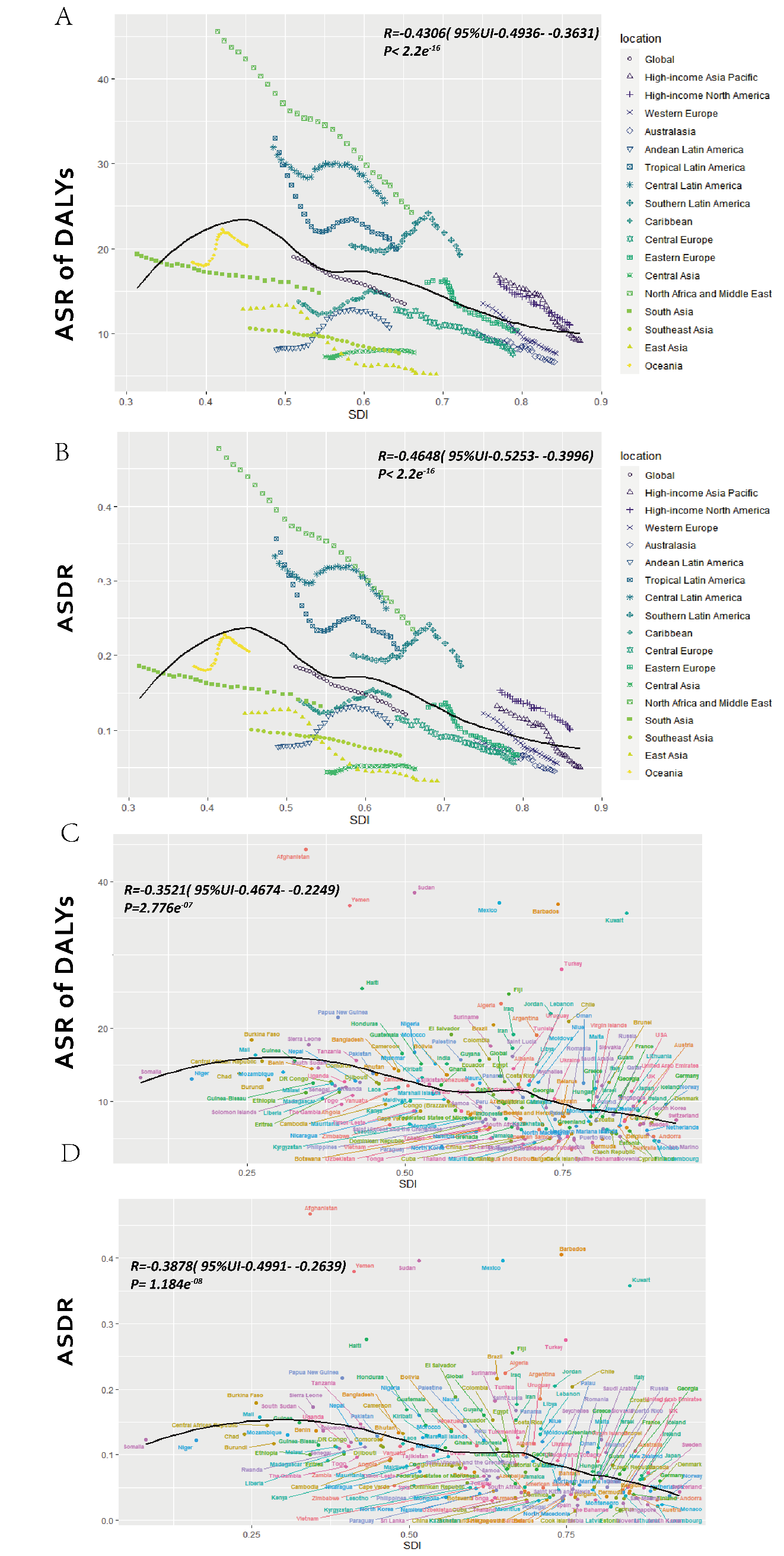


Supplementary Figure 5 The correlation between SDI and ASRs of MUCAs in 2019 at regional and national levels. (A)Age-standardized rates of DALYs and (B) age-standardized deaths rates of Male Urogenital Congenital Anomalies in 21 GBD regions from 1990 to 2019. (C)Age-standardized rates of DALYs and (D)age-standardized deaths rates of Urogenital Congenital Anomalies in 204 countries and territories in 2019. Expected values based on SDI and disease rates in all locations are shown as the black line.GBD,Global Burden of Diseases.SDI, Socio-demographic Index. DALYs, Disability Adjusted Life Years.


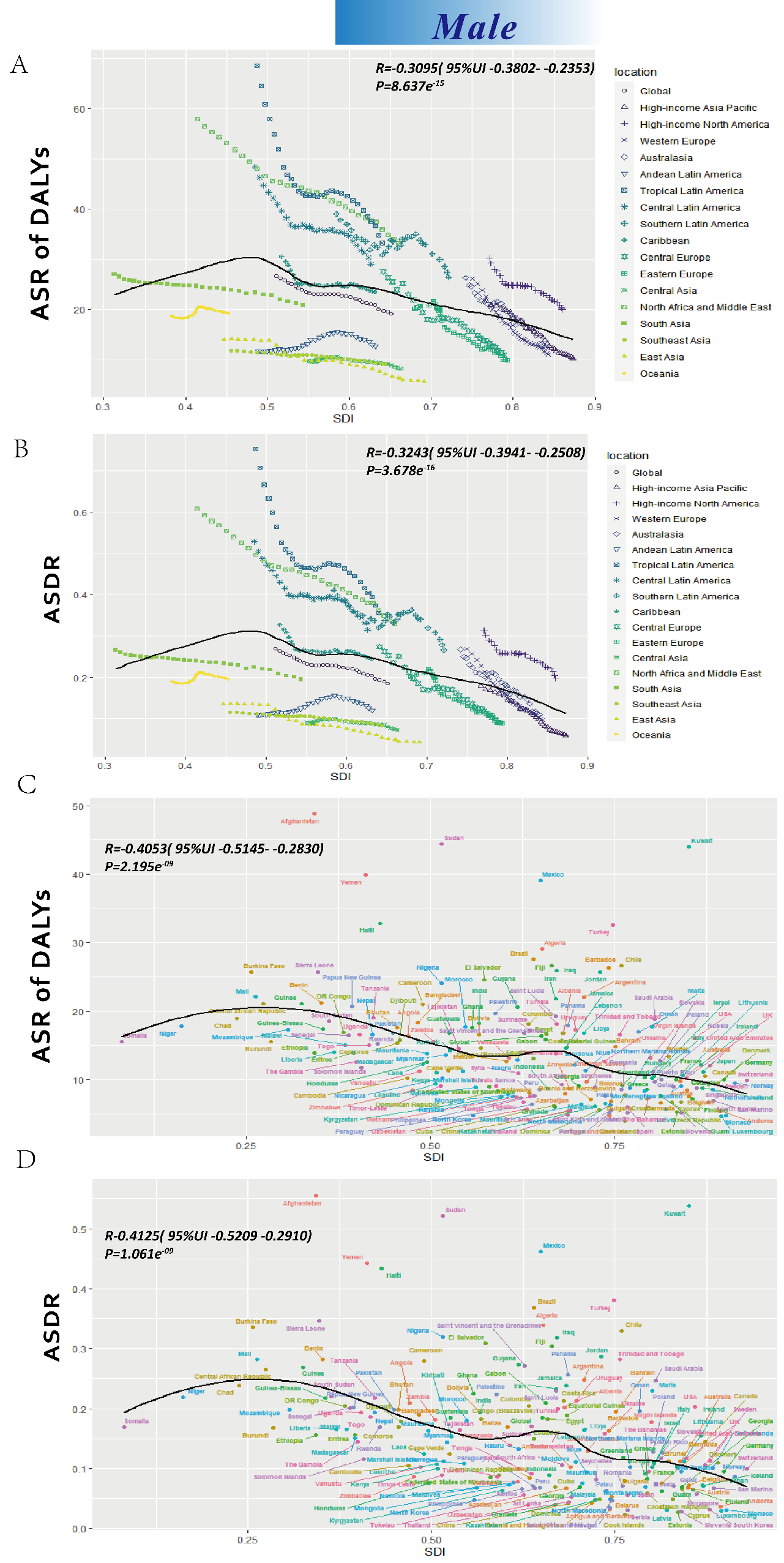

Supplement: Supplementary file 1 — Supplementary Information. [file 41598_2023_40939_MOESM1_ESM.docx]
